# Beginning of a new era of synthetic messenger RNA therapeutics: Comprehensive insights on mRNA drug design, development and applications

**DOI:** 10.3389/ebm.2025.10784

**Published:** 2025-12-12

**Authors:** Saumya Nishanga Heendeniya, Suxiang Chen, Saadia Bhatti, Qurat Ul Ain Zahra, Kamal Rahimizadeh, Bal Hari Poudel, Stephen D. Wilton, Rakesh N. Veedu

**Affiliations:** 1 Personalised Medicine Centre, Health Futures Institute, Murdoch University, Murdoch, WA, Australia; 2 Precision Nucleic Acid Therapeutics, Perron Institute for Neurological and Translational Science, Nedlands, WA, Australia

**Keywords:** mRNA therapeutics, mRNA vaccine, mRNA delivery, protein replacement therapy, mRNA design and synthesis, mRNA patents and clinical trials

## Abstract

Messenger RNA (mRNA) therapeutics have significantly transformed contemporary medicine, particularly through their role as the active component in the SARS-CoV-2 vaccine. This remarkable achievement is the culmination of extensive research conducted over many years by scientists. The widespread administration of the COVID-19 vaccine has further accelerated research into the precise therapeutic potential of mRNA technologies. Since mRNA doesn’t integrate with the host genome, the safety and versatility of mRNA-based therapeutics make them an iconic candidate in targeted therapies. Due to a surge in innovation efforts, biomodification of the molecular signatures of mRNAs like the 5′cap, untranslated regions (UTRs), and the poly(A) tail are being developed to increase translation efficacy. Recent advancements in chemical modifications, codon optimization techniques, and targeted delivery methods have significantly enhanced the stability of synthetic mRNAs while concurrently reducing their immunogenicity. Various mRNA manufacturing and synthesizing methods are investigated in this review, focusing on their scalability and limitations. mRNA therapeutic strategies can be divided into protein replacement, immune modulation, and cellular modulation. This review explores mRNA’s molecular landscape and comprehensive utility, including applications in both clinical trials and commercial sectors.

## Impact statement

The development of messenger RNA (mRNA) therapeutics is currently of paramount importance. Its successful application in SARS-CoV-2 vaccines has catalysed rapid advancements in research, demonstrating both its safety and versatility. Ongoing innovations in mRNA design continue to enhance its therapeutic potential. Beyond vaccines, mRNA is revolutionising medicine through a variety of applications, including protein replacement therapy, advanced cancer treatments such as personalised vaccines, and therapies for other infectious diseases. Additionally, it shows promise for addressing genetic and metabolic disorders. This broad and evolving adoption of mRNA display the significance and ongoing impact of mRNA technology on global health. The development of mRNA therapeutics is currently of paramount importance. Its successful application in SARS-CoV-2 vaccines has catalyzed rapid advancements in research, demonstrating both its safety and versatility. Ongoing innovations in mRNA design continue to enhance its therapeutic potential. Beyond vaccines, mRNA is revolutionizing medicine through a variety of applications, including protein replacement therapy, advanced cancer treatments such as personalized vaccines, and therapies for other infectious diseases. Additionally, it shows promise for addressing genetic and metabolic disorders. This broad and evolving adoption of mRNA displays the significance and ongoing impact of mRNA technology on global health. The article is directed towards the broader scientific community, particularly early to mid-career researchers and professionals seeking to understand therapeutic development and the commercial landscape of mRNA technology. By providing a comprehensive overview of drug design, delivery systems, commercial applications, and patent insights, the document serves as a complete resource for mRNA-related information. This makes it valuable for individuals aiming to understand the current state, future directions, and applications within this rapidly advancing field. Several review articles have examined mRNA therapeutics, particularly due to its recent popularity within the scientific community. Among these, a few stand out, notably Quin et al., “mRNA-based therapeutics: powerful and versatile tools to combat diseases,” and Lu et al., “Current landscape of mRNA technologies and delivery systems for new modality therapeutics.” The proposed review article, titled “Beginning of a New Era of Synthetic Messenger RNA (mRNA) Therapeutics: Comprehensive Insights on mRNA Drug Design, Development, and Applications,” is uniquely distinguished from the aforementioned reviews due to its explicit focus on current therapeutic and commercial applications, analyzed through published patents and clinical trial records. While all three articles provide comprehensive reviews of mRNA development, the proposed article is notable for its specific exploration of mRNA’s utility in commercial sectors and its direct incorporation of insights derived from published patents. This approach offers a distinct understanding of the intellectual property and market landscape of mRNA technologies, which is not explicitly detailed in the other two reviews. Furthermore, the proposed review article delves deeply into understanding the versatility of mRNA therapeutics, particularly in their therapeutic application to address the molecular pathogenesis of conditions underlying the aforementioned commercial inventions.

## Introduction

**FIGURE 1:**
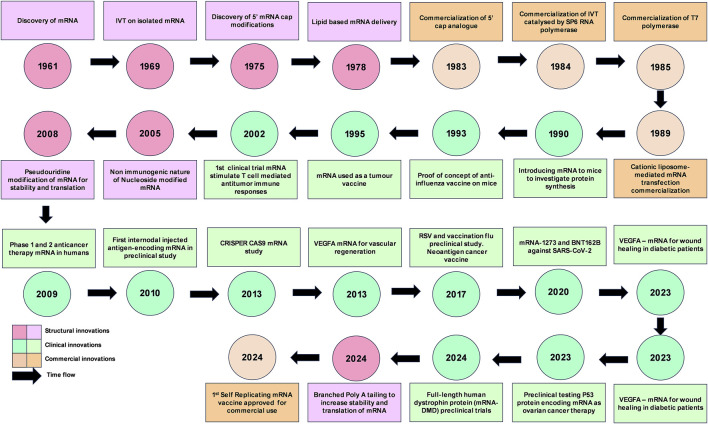
mRNA innovations timeline. IVT, *in vitro* transcription; SARS-CoV-2, Severe acute respiratory syndrome coronavirus 2; RSV, respiratory syncytial virus; CRISPR, clustered regularly interspaced short palindromic repeats; CRISPR-Cas9, CRISPR-associated protein 9; VEGFA, Vascular endothelial growth factor A; DMD, Duchenne muscular dystrophy.

Nucleic acid therapeutics are therapies formulated by deoxyribonucleic acids (DNAs) and/or ribonucleic acids (RNAs) as their key bioactive compound, inducing its therapeutic effect by modulating gene expression [1]. During recent decades, DNA and RNA-based therapies have gained significant attention as viable therapeutic options to treat a variety of health conditions including infectious diseases, cancer, neurodegeneration, metabolic disorders, and rare genetic diseases [2, 3]. Following the discovery of mRNA in 1961, scientists began to investigate its chemical properties and potential therapeutic applications (Figure 1). RNAs perform diverse yet precise functions in cellular metabolism within the human body. Approximately 75% of human genome is transcribed into RNAs [4]. RNAs can be broadly classified into 3 categories: Parasitic (self-propagating retrotransposons, viral RNA and CRISPR RNA), Regulatory and Protein-coding associated RNAs [5]. Three protein coding associated RNAs that plays a key role in protein synthesis are messenger RNA (mRNA), Transfer RNA (tRNA) and Ribosomal RNA (rRNA). mRNA plays a critical role in gene expression [4, 5]. Compared to DNA-based vaccines, mRNA therapy offers several advantages. The mRNAs’ transient nature and ability to be temporally controlled, while not integrating into genome which reduces the risk of carcinogenesis and mutagenesis, mRNA is potentially a safer alternate to traditional gene therapy. And also, mRNA only requires to be delivered into the cytoplasm of target cells without the need for direct delivery into the nucleus to initiate action, making mRNA a promising/logical therapeutic candidate [1, 4, 6, 7]. Wolff et al. introduced exogenous RNA and DNA to cells to investigate the increase in expression [8]. Later in 1993, a study conducted by Martinon et al. was the first time mRNA was used to introduce virus-specific cytotoxic T lymphocytes in animals [9]. This study paved the way for the mRNA vaccines that we currently use today [9]. The COVID-19 pandemic triggered a major shift in the use of mRNA for therapeutics, accelerating both research and public awareness [3].

Manufacturing exogenous mRNA has several benefits, primarily being more convenient, cost-effective, and final end-products not being subjected to additional regulations and scrutiny associated with genetically modified organisms [10]. However, mRNA treatments have drawbacks including delivery, stability and documented immunological side effects [11]. Exogenous mRNA is known to be cleaved by RNase, resulting in activation of Toll-like receptors (TLRs) 3, 7 and 8 inducing innate immune response that causes cytokine-mediated toxicity [6]. However, the possibility of reducing the side effects of immunogenic activity was demonstrated by Weissman and Kariko, in which modified mRNA (modRNA) was synthesized by incorporating modified ribonucleotides to reduce immunogenic activity while increasing the level of protein expression [12–14].

In fact, one of the most well-known mRNA vaccines against SARS-CoV-2 has a current count of more than 13.72 billion doses administered worldwide [15]. The present review will highlight the noteworthy uses of mRNA within the context of therapeutic and commercial applications in recent times (post 2019). Prior to this, mRNA structure, mRNA design, mRNA synthesis, molecular mechanism and therapeutic strategies will be explored.

## mRNA design and modifications

**FIGURE 2:**
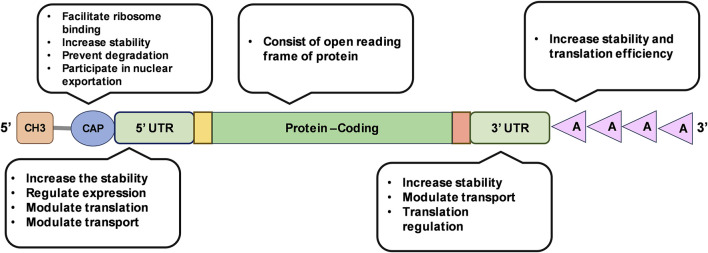
Illustration of mRNA structure and its functional features.

mRNA is a polymer of ribonucleotide building blocks, each composed of a ribose sugar, a phosphate group, and a nitrogenous base (adenine, guanine, uracil, or cytosine) [16]. Based on the principle of central dogma of life, the fundamental instructions of protein synthesis contained in DNA is expressed by transcribing the information onto mRNA. In prokaryotes, mRNA is directly transcribed from DNA and translated using ribosomes identifying the Shine-Dalgarno sequence and assembling across it in 5′UTR of the mRNA. In contrast, eukaryotes use RNA polymerase II to synthesize precursor mRNA (pre-mRNA), which is then modified to achieve the final mature mRNA. The mature mRNA is then transported to cytoplasm and translated into protein [17]. An mRNA consists of five main structural elements, namely, 5′cap, 5′untranslated region (UTR), protein coding sequence, 3′UTR, and poly adenine (A) tail (Figure 2) [10].

### Nucleotide modification

**FIGURE 3:**
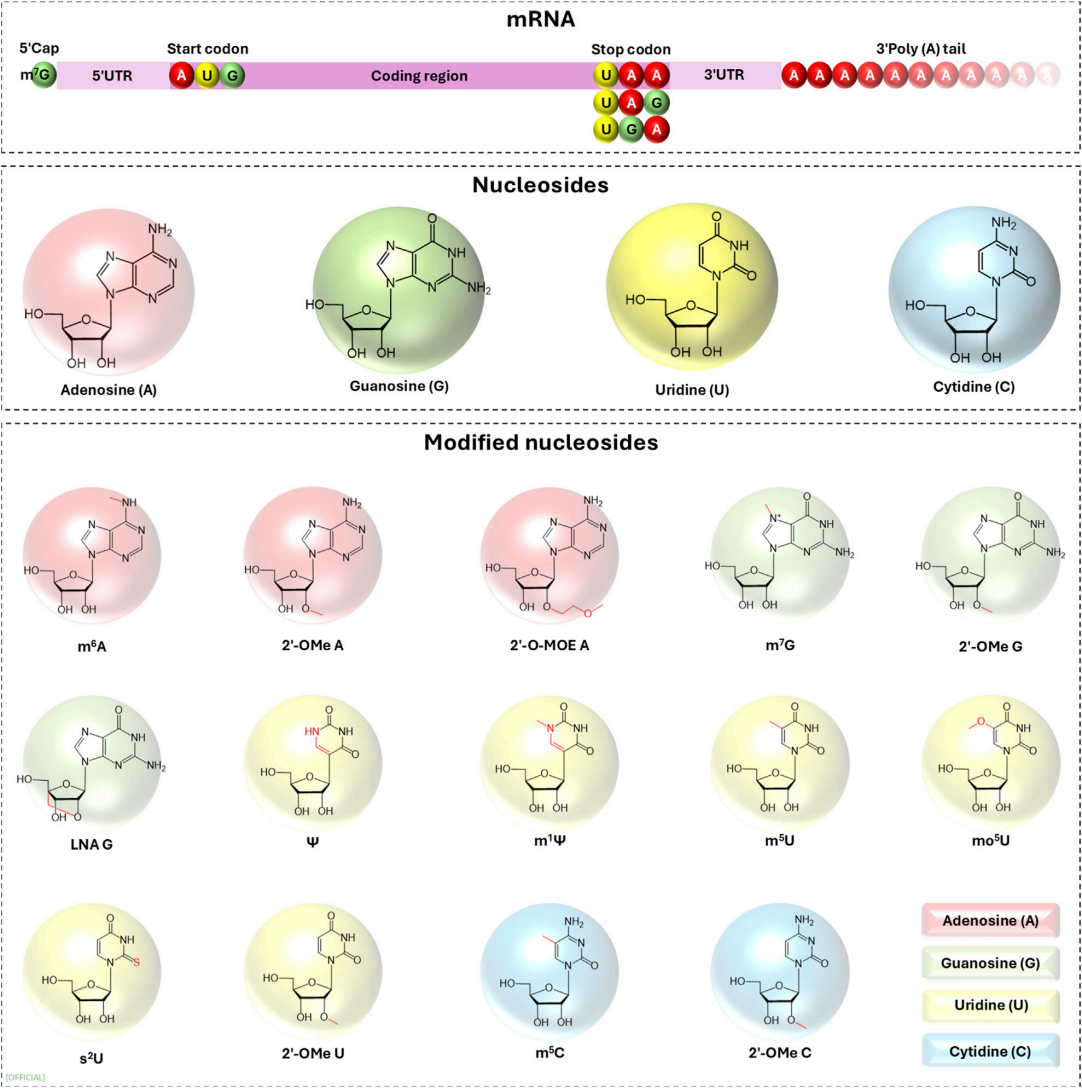
Examples of nucleoside modifications that have been investigated for constructing *in vitro* transcribed mRNA. UTR, untranslated region; m^6^A, N6-methyladenosine; m^7^G, N7-methylguanosine; Ψ, pseudouridine; m^1^Ψ, N1-methylpseudouridine; m^5^U, 5-methyluridine; mo^5^U, 5-methoxyuridine; s^2^U, 2-thiouridine; m^5^C, 5-methylcytosine; 2′-O-methyl, 2′-OMe; 2′-O-MOE, 2′-O-methoxyethyl; LNA, locked nucleic acid.

Nucleotides govern the dynamics of the expression of the genetic information encoded in the mRNA and they can be modified enzymatically or non-enzymatical (alkylation, oxidation or UV damage), to alter the biological properties of mRNA. In therapeutic and commercial applications of exogenous mRNA these modifications (Figure 3) are made in order to increase translation initiation, improve expression and reduce immunogenicity [14, 16, 18].

It is recorded that innate immune system differentiates self-originating mRNA from IVT mRNA triggering immune responses via pattern recognition receptors (PRRs). The PRRs that activate immune responses are Toll-like receptors (TLRs) and retinoic acid inducible gene I (RIG-I) like receptors (RLRs). This activation of the immune responses against IVT mRNA severely affect the translation as well as induce severe immunological side effects to the patient making IVT mRNA an unviable therapeutic pursuit. This was mitigated by the breakthrough discovery made by Weissman and Kariko who were awarded the Nobel prize in 2023 [19]. Weissman and Kariko explored the possibility of using modified nucleotides (m^5^C, m^6^A, m^5^U, s^2^U or pseudouridine) in IVT mRNA to reduce immunogenicity of IVT mRNA. They discovered that using modified nucleotides (m^5^C, m^6^A, m^5^U, s^2^U or pseudouridine) in IVT mRNA, ablates the activation of TLR3, TLR7, TLR8 and TLR9 leading to a significant reduction in the production of type I interferons (IFNs) and pro-inflammatory cytokines compared to unmodified IVT mRNA (Figure 3) [20, 21]. According to Weissman and Kariko, translation of Ψ-containing transcripts was 4-5-fold greater than that of unmodified transcripts in wild-type mouse embryonic fibroblasts (MEFs), yet in PKR knockout (PKR^−/−^) MEFs, the extent of translation of Ψ-modified mRNA was not different from that of unmodified mRNA. Thus, displaying that Ψ-mRNA translational increase is dependent on PKR activity [6, 14]. In the following year they identified that IVT mRNA containing uridine has a higher tendency to activate RNA-dependent protein kinases (PKR) which then phosphorylates translation initiation factor 2-alpha, inhibiting translation. The activation of PKR is limited in IVT mRNA containing pseudouridine (ψ) modification, thus increasing the translation relative to unmodified IVT mRNA [14]. During *in vivo* studies, 8-week-old BALB/c mice (weighing 18–23 g) was treated with 3.0 µg of Ψ-modified mRNA and unmodified mRNA it was observed that, unmodified mRNA induced high levels of IFN-α in serum while Ψ-modified mRNA did not induce higher level of IFN-α, 12-fold higher protein expression was observed [6].

Following the Kariko paradigm, Andries et al. identified that N1-methylpseudouridine (m^1^Ψ) alone or in combination with 5-methylcytidine (m^5^C) modification, displayed comparatively higher translation rates and increased stability. The modifications have shown a 13-fold increase in expression in single modified mRNA and 44-fold increase in double modified mRNA expression in mammalian cells and mice. They observed that (m^5^C/)m^1^Ψ-modified mRNA triggered a reduced innate immune response compared to (m^5^C/)Ψ-modified mRNA post-transfection making it a new benchmark for mRNA modification [22]. Following these inventions, in 2020 Pfizer-BioNTech m^1^Ψ-modification was used in SARS-CoV-2 mRNA vaccine [23]. Like Ψ-uridine, m^1^Ψ-uridine also evades processing by RNase T2, PLD3, and PLD4. But N1- m^1^Ψ-uridine is capable of directly activating TLR8. This selective activation activating TLR8 pathway without broader immune activation is leveraged in vaccine development and other immunotherapeutic strategies [24].

However, Mulroney et al. discovered that N1 methyl Ψ-modification (m^1^Ψ) causes +1 ribosomal frameshifting during IVT mRNA translation leading to the unintended creation of altered proteins with off-target immune responses. Even though there is no noted adverse effects arising from mistranslation, this exhibits room for improvement in mRNA design optimization [25].

### Codon and sequence optimization

Codons are three-letter nucleotide sequences that correspond to amino acids and stop signals during protein synthesis. There are 64 codons that are specific to 20 amino acid building blocks and three stop codons. This nature of degeneracy is a feature of genetic code in all organisms. Apart from degeneracy, other key features of codons are being non-overlapping, non-ambiguous and universal among all living organisms [26]. The nature of degeneracy of genetic code creates the existence of synonymous codons that encode the same amino acid [27]. The rate of decoding these synonymous codons are biased, which is evident by the disproportionate representation in certain codons over other types in the transcriptome [28]. The codons with less than 1% frequency in the genome is known as a rare codon. It is observed that the cognate tRNA associated with the rare codons are less abundant in organisms and are referred to as rare tRNAs [29]. The efficiency of the selection of cognate tRNA from the cytoplasmic tRNA pool determines whether it is optimal or not. Hence, codon optimality is governed by the tRNA availability and the stochastic kinetics of the ribosomes [28]. A genome-wide analysis of mRNA decay rates conducted by Presnyak et al. revealed that mRNAs composed predominantly of optimal codons are on average more stable than the mRNAs containing predominantly non-optimal codons [30]. On the contrary, Saikia et al. study identifies that rare codons promote selective protein synthesis rather than affecting mRNA stability in mammalian systems during amino acid (aa) starvation. They identified that genes enriched in non-optimal codons [like ubiquitin-proteasome system (UPP)] maintained or even up-regulate their translation efficiency while ribosomal protein genes which are rich in common codons, experience translational suppression during aa starvation. The underlying mechanism involves the selective charging of rare tRNA isoacceptors which maintains translation, while abundant tRNA isoacceptors are depleting during starvation. Experimental validation was conducted using FLuc (firefly luciferase) reporters, with rare codons in UPP gene sequences enable sustained translation during amino acid starvation. Yet when rare codons were changed to common codons, the protein levels have been reduced. The team further discovered that the above translational effects had occurred while total mRNA levels were unchanged, indicating that codon optimality regulates protein synthesis at the translational level rather than through mRNA degradation or stabilization [31].

In molecular therapeutics, codon optimization is used to increase the expression and stability of recombinant proteins by avoiding the rare codons which can restrict protein synthesis. It has been identified that species with large population sizes and highly expressed genes are most likely to be encoded with codons with abundant cognate tRNA. And usage of codons with more abundant cognate tRNA significantly increases the protein expression more than 1000-fold [27]. However, this correlation is not well-established in humans. As study conducted by Parmley and Hyynen (2009) shows that humans have clusters of codons with low abundance cognate tRNA (rare cognate tRNA), hinting at novel mechanism of tissue-specific transcription regulation [27, 32]. It has been also identified that affecting codon optimality by having non-optimal codons is directly proportional to the instability of the mRNA [28]. Following the above revelations, Wang et al. engineered rare codon devices to manipulate the expression levels of proteins in *E. coli.* In their study, codon devices were utilized to manipulate four genes in fatty acid synthesis pathway, where they successfully increased the fatty acid yield by approximately two-fold [33].

The COVID-19 pandemic has accelerated the research in codon optimization where scientists are now exploring a variety of methods to design more stable mRNAs. Zhang et al. used classical lattice parsing in computational linguistic to develop an *in silico* algorithm of Linear Design to optimize structural stability and codon usage in mRNAs. Using the Linear Design algorithm, they developed novel designs for SARS-CoV-2 spike protein and varicella-zoster virus glycoprotein E protein. Relative to benchmarks, these novel mRNA designs have exhibited 2.3 - 2.9 fold increase in protein expression and a 128-fold increase in antibody response for vaccines, demonstrating the importance of UTR engineering and codon optimization [34]. Diez *et al*., has developed the algorithm known as iCodon which is a machine learning model trained with mRNA stability from zebrafish, *Xenopus* embryos, human cell lines, and mouse embryonic stem cells, to predict mRNA stability based on codon composition. During the study the team has restored the function of optimized variants of the slc45a2 gene, injected into albino zebrafish embryos, successfully restored pigmentation. Diez and the team has also improved the fluorescence intensities of AausFP1 protein in human 293T cells. iCodon-optimized variant had displayed 4-fold increase in fluorescence intensity compared to the original AausFP1 [35].

Research has displayed that maximal codon optimization doesn’t always yield the best translational results, over- optimization could lead to synthesis of functionally and structurally altered proteins [27]. Therefore, the current consensus among scientist is to approach codon optimization holistically taking into consideration of evolutionary pressure and natural selection [36].

### 5′Cap

**FIGURE 4:**
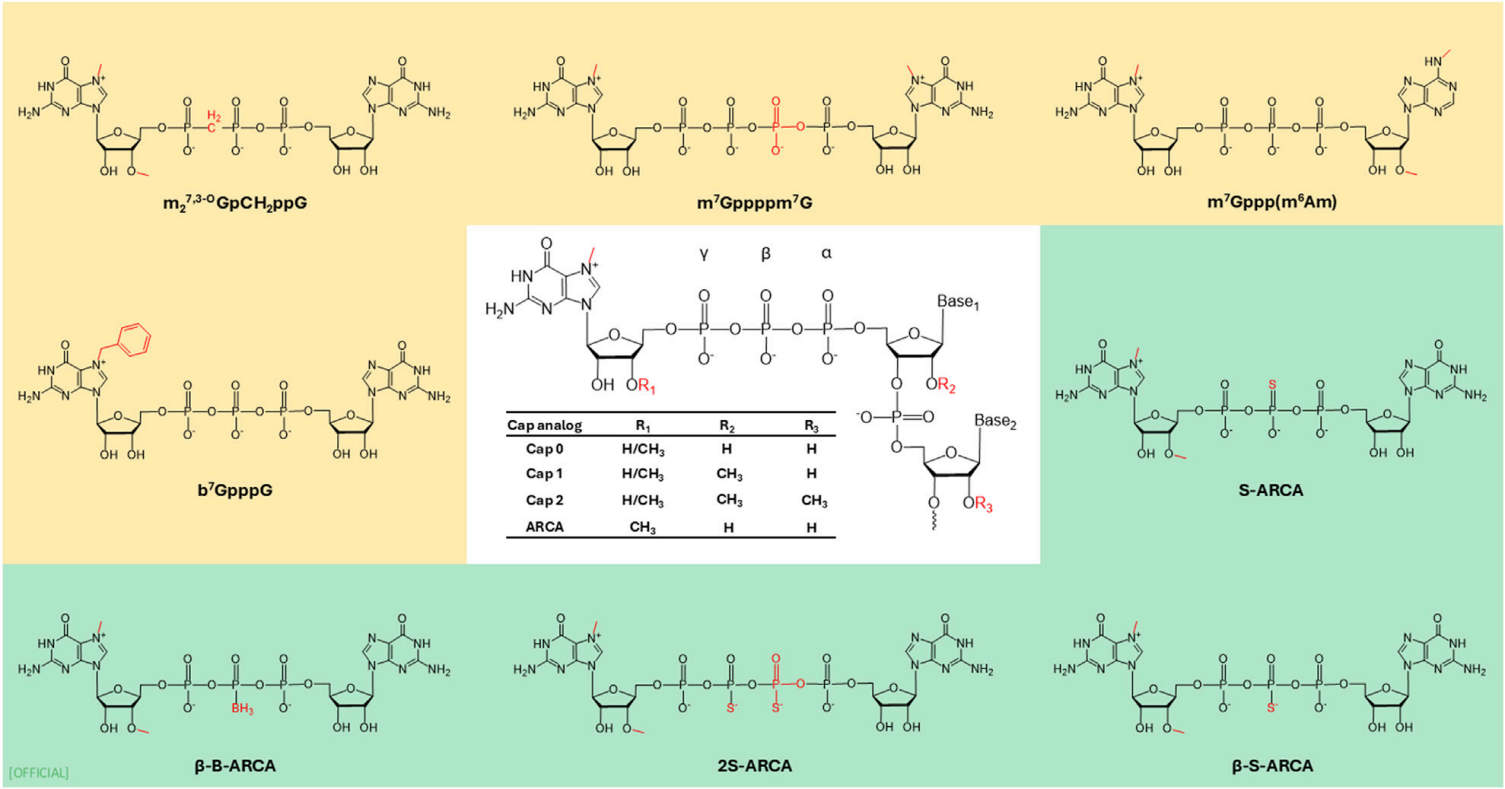
Examples of 5′cap modifications. ARCA: anti-reverse cap analog.

The 5′end of mRNA possesses a unique structure known as a cap, which is composed of a 7-methylguanosine (m^7^G) linked to the mRNA via 5′-5′ triphosphate bridge (ppp). This particular cap is identified as “cap 0.” The 5′cap plays a fundamental role in protecting eukaryotic mRNA from premature exonuclease cleavage (Dcp 1/2) and assists mRNA transport and cap-dependent translation [37]. In the pre-mRNA, the 5′cap facilitates polyadenylation, nuclear export, and splicing by recruiting specific protein factors [38]. In the mature mRNA, it recruits eukaryotic translation initiation factor 4E (elF4E) via hydrophobic cation–Π interactions and electrostatic negative interactions of the triphosphate bridge. The cap is essential for the self- and non-self-discrimination of cellular RNAs to initiate innate immune responses against foreign RNAs. The mechanics of innate immune activation is by retinoic acid-inducible gene I receptor (RIG-I). The immunogenicity of cap zero is reduced by 2′-O methylation of the ribose sugar of the first (R1) or second (R2) nucleotide (Figure 4), creating cap 1 and cap 2 [39]. Cap 1 structure characterized by and often 2′-O-methylated, 7-methylguanosine linked to the first transcribed nucleotide [40, 41]. Despic and Jaffrey (2023) discovered that C1 is modified to C2 in the cytoplasm by cap methyltransferase 2 (CMTR2) in mRNA with a longer half-life [42]. The dual methylation in cap 2 enhances the stability and protein expression in transcripts by reducing the binding ability of the cap one mRNA to RIG-I [42].

In cells, 5′Cap is introduced to mRNA by three sequential enzyme reactions [43]. *In vitro* transcribed (IVT) mRNA undergoes a capping reaction, which can be executed either post-transcriptionally or co-transcriptionally. Post-transcriptional capping is commonly done by a recombinant viral capping enzyme, such as vaccina virus-capping enzyme (VCE). VCE catalyses the capping reaction where standard eukaryotic m7GpppG structure is added to mRNA. Co-transcriptional capping is a reaction where a synthetic cap is added during transcription. This method provides versatility of using different cap structures yet has the drawback of potential synthesis of partially capped mRNA. Partial capping could take place due to, competition with GTP in IVT as well as cap binding protein hindered by incorrect orientation of the cap. To mitigate this, the anti-reverse cap analogue (ARCA: 3′-O-Me-m7G(5′)ppp(5′)G) was developed [19, 44]. Compared to ARCA which is a Cap 0 structure, CleanCap® on the other hand is co-transcriptional cap with cap 1 structure has mitigated with drawbacks of Cap 0: ARCA Cap, while being 94% capping efficiency [45]. A noteworthy method in increasing the rate of translation involves replacing the m7G with 7-benzylated guanosine. It has been reported that attaching the m7G with another m7G via tetraphosphate (m7Gppppm7G) increases the affinity of the mRNA with elF4E compared to the natural analogue, thereby increasing the translation by two fold [104]. Furthermore, Grezela et al. have modified this base cap by N2 modification to further increase translation and stability [46]. This concept of synthetically modified caps is seen in FDA-approved SARS-CoV-2 vaccines, with a cap analogue 7mG[5']ppp[5']NlmpNp increasing translation rates [104]. Pfizer-BioNTech and Moderna utilize cap 1 analogue in their vaccines [47]. However, Pfizer-BioNTech uses synthetic chemical process, while Moderna’s Cap 1 analogue is obtained through a chemical reaction catalysed by Vaccinia capping enzyme and Vaccinia 2′-O-methyltransferase [47, 48]. Warminski et al., (2024) have demonstrated that combination of trinucleotide capping with modifications in phosphate groups in cap structure is a potential avenue to develop novel high quality synthetic mRNA cap structures [43]. To further improve translation efficiency Senthilvelan et al., synthesised a novel locked nucleic acid (LNA)-modified trinucleotide cap analogue m7(LNA)G(5′)ppp(5′)AmpG, which has increased the translation efficiency of mRNA *in vitro* by factor of 5 relative to the ARCA-capped mRNA. By LNA modification and the C3′-endo (N-type) conformation in the LNA structure has increased intracellular stability, resulting in increased translation efficiency [49]. Another promising development is photocaged 5′cap analogues in the context of cap structures. This specific cap analogue serves to regulate expression by spatiotemporal control, thus further refining the targeting and activation of genes. Klöcker et al., have developed this 5′ Cap with photo-cleavable groups called “Flash Caps” which inhibit translation by blocking the elF4E binding. During *in vitro* studies when exposed to laser irradiation cleaving the Flash Cap group restores Cap 0 translation. This development creates a novel approach to regulate expression by spatiotemporal control [50]. Tarbashevich et al., has displayed light-controlled protein expression in Zebrafish embryo *in vivo* model, where they were able to induce the expression of fluorescent proteins for cell labelling, toxins for targeted cell ablation, and morphogens like Bmp2b and β-catenin to influence embryonic development [51]. The advancement in molecular structures of 5′cap not only enables production of more stable mRNA with higher expression levels, but it has also created new avenues to regulate gene expression.

### The untranslated region (UTR)

UTR is non-coding sequences located at 5′and 3′of the coding regions of the mature mRNA. Current knowledge stipulates that UTRs regulate localization, translation, and stability of mRNAs. It has been identified that the length, start-site consensus sequence, secondary structures, upstream open reading frame, upstream the canonical AUG, internal ribosome entry site (IRES) and sequences that bind regulatory proteins present in 5′UTR are responsible for the regulation of mRNA translation rate [52]. The 5′UTR plays a crucial role in initiation of translation by recruitment of the ribosome and initiation factors to assemble the pre-initiation complex. The 5′cap identifies and recruits elF4A initiation factors essential for the translation. In addition, 5′UTR in some instances contains internal ribosome entry sites which recruits ribosome and initiate cap-independent translation. The 5′UTR also plays a crucial role in the regulation of transcription by interacting with proteins such as iron regulatory proteins. These proteins bind to specific structures within the 5′UTR, known as iron-responsive elements (IREs), thereby modulating translation in response to varying iron levels. [53, 54]. The 3′UTR is integral to the regulation of mRNA translation and its stability. In eukaryotes the elF4G interacts with poly (A)-binding proteins which bind to the poly (A) tail of the mRNA creating a circular loop, and this conformation is believed to promote efficiency [55]. It has been identified that 3′UTR plays a role in mRNA localisation as well [56].

It is known that some UTRs present in human genome promote higher translation rates than other UTRs. From a therapeutic standpoint, UTRs are key focus areas in research and development for maximizing translation rate and further stabilizing mRNA constructs. A study conducted by Asrani (2018) highlighted that combinations of different 5′UTRs of certain genes with 3′UTRs of other genes have introduced variation of expression in IVT mRNA. According to this study, the 5′UTRs of complement factor 3 (C3) and cytochrome p4502E1 (CYP2E1), consistently exhibit elevated expression of proteins in IVT mRNA [57]. Research conducted by Linares-Fernández et al. demonstrated that the 5′and 3′UTRs of ß-globin enhance translation efficiency. The study also indicated that using two 3′UTRs in tandem produces higher expression relative to using one ß-globin 3′UTR [58]. This effect of higher expression based on using repeated 3′UTR was later shown to be cell type-dependent and single 3′UTR has displayed higher protein expression that multiple tandem 3′UTRs [104]. The closely related field of bioinformatics has contributed to the strides made in UTR engineering via deep learning and *in silico* techniques. The development of novel UTRs contributes to higher expression and stability of mRNAs. Studies conducted by Cao et al. and Castilo-hair et al. show novel methods of developing optimized synthetic UTRs using *in silico* techniques, revolutionizing mRNA therapeutics [59, 60].

### Poly(A) tail

The Poly(A) tail plays a critical role in translation initiation and the control of mRNA translation efficiency. According to the circular polysome model, translation initiation commences when eIF3 and met-tRNA bound 43s ribosome subunit forms a complex with 5′cap bound eIF4E and 3′poly(A) tail bound poly(A) binding protein (PABP) brought together by eIF4G. Translation efficiency has shown to be directly related to the length of the poly(A) tail. Most mammalian mRNAs consist of 200–250 adenine units in their poly(A) tail. Poly(A) tails also play a significant role in preserving the integrity of mRNA by reducing its length throughout the life span of the mRNA sequence. This reduction will continue until the length of the poly(A) tail has only 10–15 units left [54, 61–63]. Although this shortened poly(A) tail is unable to allow mRNA to associate with PABP or initiation factor eIF4E, preventing mRNA expression, the shortened tail associates with exoribonuclease 1 (XRN1), exoribonuclease RRP6, Dcp1p, Dcp2p and/or the cytoplasmic Lsm1p-7p followed by de-capping and subsequent degradation [64].

Several researchers have evaluated the effect of the length of poly(A) tail on translation efficiency. Peng et al., explored cell free *in vitro* translation system with Hela S3 cell cytoplasmic extract system in addition to *in vitro* translation using LM (tk-) cell transfection to investigate the translation efficiency relative to poly(A) tailing. This revealed that increase of poly(A) tail length from 0 to 98 adenosine residues progressively increased the translation from 1 to 25 fold [61]. According to Elango et al., increasing the poly(A) tail length of FLuc mRNA from 0 to 60 adenosine residues markedly increased FLuc expression in UMR-106 cells and this expression reduced after the length of 100 adenosine residues. Further findings in the study observed that mRNA translation was reduced for mRNA sequences containing heterologous sequences after the poly(A) tail [62].

The research conducted by Holtkemp et al. demonstrated that a length of 120 adenosines is optimal for translation in human dendritic cells [65]. This discovery was confirmed by Oh and Kessler, Avci-Adali et al. and Preskey et al. who have conducted studies of synthetic mRNA with poly (A) tail containing 120 adenosine residues where protein was successfully expressed *in vitro* [66–68]. S. Linares-Fernández et al. analysed variable of poly(A) tail lengths corresponding to their protein expression and observed that poly(A) tail containing 148 adenines increased the mRNA stability as well as expression in HeLa cells after 24 h post-transfection. However, the impact of poly(A) size has been recently re-evaluated, with recent studies demonstrating that longer tail (300 nucleotides) sequences are more optimal with novel mRNA designs [58]. The inconsistent research findings underscore the necessity for further investigation into the role of poly(A) tail length in mRNA kinetics. Grandi et al., examine the potential causes of these inconsistencies by decoupling mRNA degradation and translation in relation to poly(A) tail length. In this study, a library of identical mRNAs with varying poly(A) tail lengths was created, and mRNA kinetics were analysed in human embryonic kidney cells. The study demonstrates that while poly(A) tail length influences degradation, it does not directly determine the translation rate [69]. This research offers a potential explanation for the variation in translation efficiency associated with different poly(A) tail lengths. Recently Chen et al. developed a novel mRNA design with branched poly(A) tail. This multi-tailed mRNA encoded FLuc as the target protein while using RLuc (renilla luciferase) as the standard. HeLa cells were transfected using this mRNA and had displayed 4.7 to 19.5-fold increase in protein expression in 24, 48, and 72 h’ time intervals [70], showcasing the potential of promising developments focused on poly(A) tail structures in the mRNA design landscape.

## Exogenous mRNA manufacturing

Before the COVID-19 pandemic, mRNA synthesis was a niche technology, and the scale of synthesis was relatively small. The COVID-19 pandemic saw a paradigm shift in the development and synthesis of mRNA for medical and research purposes. mRNA synthesis and manufacturing were upscaled to meet the surge in demand for the COVID vaccine. In 2021, mRNA synthesis became an urgent need due to the SARS-CoV-2 vaccine with a global demand of 1-2 billion doses. High volume exogenous synthesis of mRNA is mostly conducted using three methods: chemical, recombinant or enzyme-based systems [71].

Chemical synthesis (CS) of RNA utilizes phosphonamidite chemistry and solid phase support for continuous synthesizing RNA of less than 100 nucleotides (nt). The main limitation of solid phase chemical synthesis is size which is <100 nt. To synthesis longer RNA, short RNAs should be ligated together. This limits the scalability of this method of synthesis [71]. Yet CS has the distinct advantage of its ability to introduce site specific modifications like 2′-F modifications in to mRNA [72]. Abe et al. and Husseini et al. have developed a short mRNA therapeutic treatment known as minimal mRNA vaccine. According to Husseini et al., chemically synthesised minimal mRNA vaccine has shown promising results against melanoma with tumour inhibition and upregulation of cytokine expression after 22 days of treatment [73, 74].

Recombinant synthesis system (RSS) follows similar biomechanical method to recombinant protein production. In RSS, the mRNA sequence is amplified using either *in vitro* amplification or using *in vivo* amplification (with host cell like *E.coli*). Curry et al., has developed an *in vivo* mRNA synthesis platform using *E.coli* where *E.coli* are genetically engineered to use the bacteria’s mRNA synthesis system to synthesise synthetic mRNA. This novel platform has increased the product yield upto 40-folds compared to the unmodified *E.coli* expression systems [75]. RSS with *in vivo* amplification allows for the potential of large-scale production without a size limitation like CS, degradation of the transcribed mRNA by host nucleases and inability to use chemically modified nucleotides in the synthesis process are major draw backs of the system. Therefore, the enzyme-based system of *in vitro* transcription is considered as the gold standard method for the large-scale mRNA production [71, 76].

*In vitro* transcription (IVT) uses a linearized plasmid DNA (pDNA) template, or a DNA template amplified by polymerase chain reaction (PCR) for mRNA synthesis. The core enzyme that is used in this mechanism is bacteriophage RNA polymerase (T7, T3 or SP6), which recognizes the promoter in the DNA. Once the synthesis is completed, the dinucleotide m^7^GpppG as the structural homolog of endogenous Cap-50 and a poly(A) tail are added [17]. The main drawback of IVT system is the necessity of sequence-specific promoter for transcription initiation which limits terminal heterogeneity and the non-specific runoff in mRNA. Recent advancements in deep learning and sequencing has provided researchers with tools like synthetic libraries to optimize 5′ UTR sequences. Inclusion of human hydroxysteroid 17-beta dehydrogenase 4 genes’ 5′ UTR region in an mRNA vaccine candidate (CV2CoV), has enhances the translational capability of the mRNA [77]. Currently, IVT is the technology that is used for the synthesis of cost-effective large-scale clinically used mRNA following Good Manufacturing Practices (GMP) specification [66, 67, 71].

IVT system can be divided into three major stages: template amplification, *in vitro* transcription and purification. Depending on the scale (micro scale, bench scale, commercial scale) of mRNA synthesis, different techniques and equipment are employed based on the methods discussed previously [71]. In the micro scale synthesis, mRNA encoding linearized DNA is amplified using PCR, which is then used as the template for capped IVT reaction. The product is then purified using silicon-based columns such as Amicon Ultra-15 centrifugal filter units(30K membrane) (Millipore), RNeasy (Qiagen) and MEGAclear™ transcription clean-up kit (Thermofisher scientific) [66, 78]. At both bench and commercial scales, amplification involves producing mRNA from the DNA encoded in the plasmid, which is replicated using transformed bacterial cultures. On the bench scale, these cultures are typically grown using a batch processing protocol. In contrast, at the commercial scale, the transformed bacteria are propagated in bioreactors using a semi-batch processing approach. The extracted amplified template is used for IVT reaction, where the mRNA is synthesized and subsequently purified. A commonly used purification in bench scale is lithium chloride precipitation. On a commercial scale, techniques like ion exchange chromatography (IEC), ion-pair reverse chromatography (IPC), diafiltration using tangential flow filtration, and affinity chromatography are used. [3, 66, 71, 76]. The purification of modified mRNA is crucial for its functional activity. Contaminants present in in vitro transcribed RNA, such as double-stranded RNA, can induce immune responses, including the activation of type I interferons (IFNs) and proinflammatory cytokines. Therefore, the removal of these contaminants is imperative. The comprehensive study conducted by Karikó et al. demonstrates that high-performance liquid chromatography (HPLC) is one of the most effective purification methods. This technique yields mRNA that does not induce IFNs and inflammatory cytokines and is translated at levels 10- to 1000-fold higher in primary cells compared to poorly purified mRNA [78].

One drawback of using bacteria-based plasmid vectors for amplification is that researchers have noticed spontaneous deletions in the poly A tail. A study by Elong et al., tested different bacterial strains—DH5α, TOP10, GM2163, XL1-Blue, and XL10—to grow plasmids containing mRNA templates [62]. They found that all strains showed some reduction in poly A tail length, but XL1-Blue preserved the longest tail [62]. To improve the integrity of in vitro-transcribed (IVT) mRNA, scientists are developing new methods. He et al., created an advanced technique using a modified T7 polymerase and optimized IVT conditions. This approach achieved over 91% mRNA integrity, significantly improving on existing protocols [70].

## mRNA encapsulation and delivery

Given the significance of mRNA and the first successful mRNA vaccine platform against COVID-19, the era of emerging disorders surged the escalating demand for targeted delivery systems for mRNA encapsulation and delivery [79–81]. As with other nucleic acids, mRNA is a macromolecule unable to cross evolutionary complex barriers (lipid bilayer and blood-brain barrier) making targeted delivery toward hard-to-reach cells a paramount challenge [82]. With the molecular weight of >10^6^ g/mol, and electrostatic repulsion of negatively charged mRNA further impedes the penetration across the formidable cell membrane barrier [79, 82]. Moreover, without a delivery vehicle, the cell penetration potency of naked mRNA is lower [80]. Additionally, the shorter half-life together with enzymatic degradation by endonuclease, 5′and 3′exonucleases, raises further instability and immunogenicity concerns [80]. All these extracellular and intracellular barriers collectively necessitate establishing a delivery vector for the targeted release of mRNA in both *in vitro* (cell-based models) and (animal models) *in vivo* systems.

In past years, various physical methods have been deployed to facilitate mRNA uptake into the cells, primarily through transfection methods that introduce mRNA into the cytoplasm. Electroporation is one of the traditional methods with vast clinical applications including cancer immunotherapy, gene editing, and protein replacement. The principle of this method is based on nucleic acid delivery via enhanced membrane permeability through electric field induction [83]. Similarly, a gene-gun (a gold nanoparticle encapsulated mRNA vector) is one of the primitive mRNA delivery technologies for introducing mRNA into the targeted cell using a pressurized carrier gas [84]. Another method is microinjection-a type of mechanoporation that uses a micropipette/nanopipette for the direct injection of nucleic acid into intracellular space [85]. Microfluidic transfection is another innovative device for robust mRNA transport to chimeric antigen receptors expressing immune cells and NK cells. The mode of delivery is based on the principle of volume exchange for convective transfection (VECT) [86]. VECT devices are more advantageous over traditional electroporation platforms, especially for co-transgene expression, owing to their robustness, simplicity, and low genotoxicity [86].

Unfortunately, at present most cancer immunotherapies as well as prophylactic vaccines rely on local administration. With an emerging era of effective mRNA therapeutic delivery, the systemic route of administration is the major requirement but poses several targeted delivery challenges (Figure 6) [87, 88]. To circumvent this problem, encapsulation vectors-viral and non-viral-based systems have emerged harnessing the biomimetic potentials of nanobiotechnology [88]. Nonetheless, nanoparticles must be designed to mitigate the evolutionary cell barriers that hamper the targeted delivery of mRNA drugs to their main site of action. Upon systematic delivery, nanoparticle-based vehicles must escape endo-lysosomal vesicles, avoid renal clearance, and phagocytosis by circulating immune cells (Figure 6) [89]. They must also avoid non-specific plasma protein binding and be able to penetrate across-capillary endothelial barriers to reach their destination-the cytosol, for releasing the mRNA cargo for translation to occur [88]. Only about 2% of the nano-encapsulated drugs escape the endosomal entrapment, which has been incredibly challenging for macromolecules (>5 nm) to permeate the capillary endothelium; therefore, drugs are accumulated in the spleen and liver leading to unwanted toxicities. Finally, after reaching the dense extracellular matrix, the high osmotic pressure can impede fibrous proteins [90].

After reaching the extracellular matrix, the delivery vehicle must traverse a network of fibrous proteins and polysaccharides to reach the target cell membrane [1, 90]. Hence, the widespread clinical application of mRNA therapeutics faces a significant hurdle in finding safe and effective delivery systems.

Viral vs. non-viral vectors: While viral vectors like lentiviruses, adeno-associated viruses, and the Sendai virus have shown the capability to deliver nucleic acids systemically, their use can be hampered by unwanted immune responses [91, 92]. Importantly, many of these delivery materials have benefited from two decades of research of non-viral short-interfering RNA (siRNA) delivery [90]. Although siRNA differs from mRNA in being double-stranded, more rigid, and smaller (about 42 bases compared to over 1,000 bases for the average mRNA), they share the same chemical building blocks and require delivery to the same cellular destination, namely, the cytoplasm [90].

### Non-viral vectors for mRNA delivery-clinical applications

The use of viral and synthetic vectors in nucleic acid delivery has revolutionized the field of genetic nanomedicine, notably contributing to the rapid development of highly effective COVID-19 vaccines (mRNA-based delivery platform) [7]. This breakthrough has opened new horizons for targeted nucleic acid delivery in nanomedicine. Both viral and synthetic vectors come with their own sets of advantages and disadvantages. Synthetic vectors offer the distinct advantage of limitless synthetic capabilities. Among nonviral vectors, lipid nanoparticles (LNPs), comprising four components, have emerged as the leading choice for mRNA delivery, notably utilized by Pfizer and Moderna in their COVID-19 vaccines [93].

### Liposomes and lipid nanoparticles (LNPs)

**FIGURE 5:**
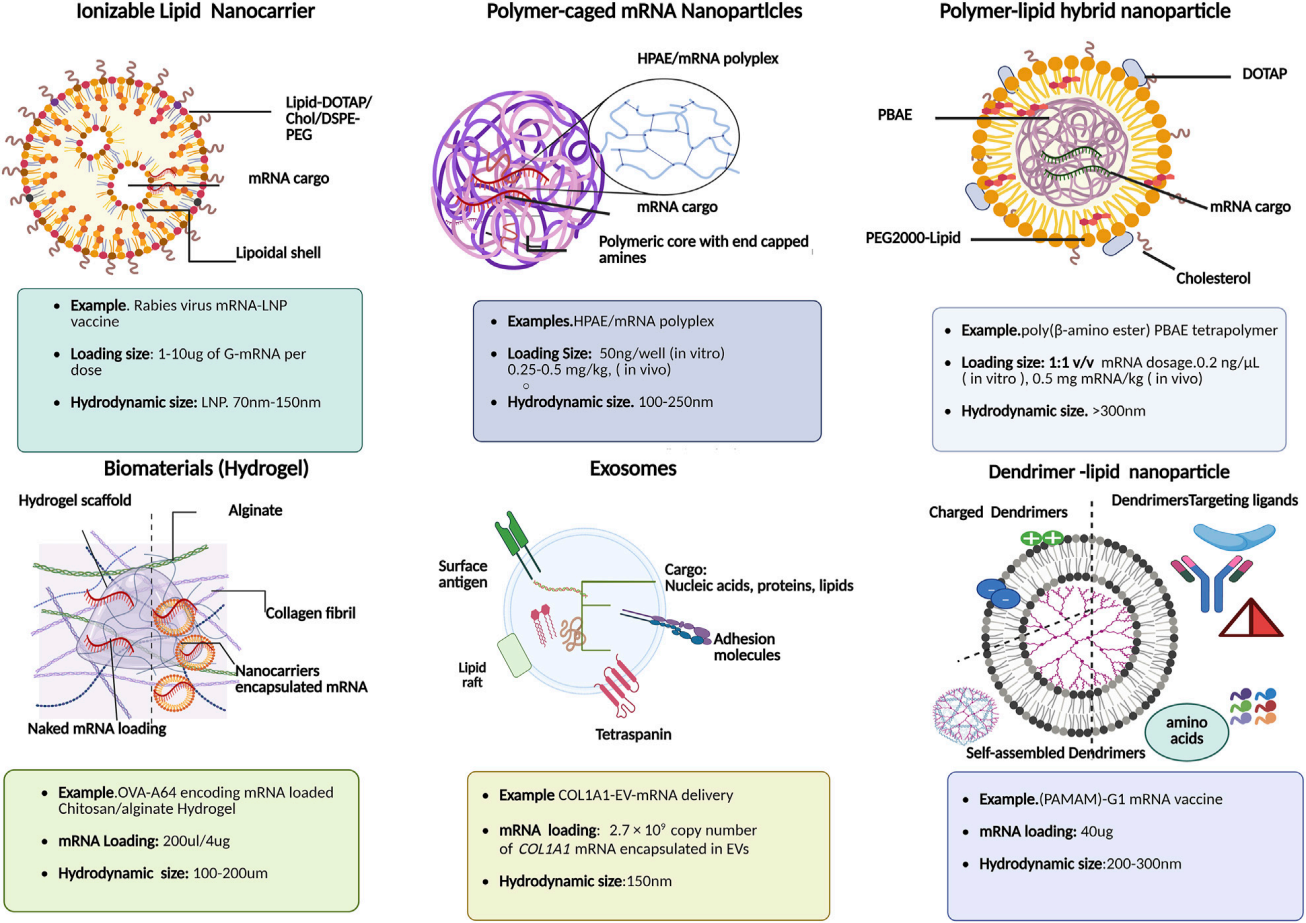
Schematic illustration of various functional mRNA-loaded nanocarriers: their composition and physiochemical features for downstream clinical applications in mRNA therapies.

Similarly, to circumvent the challenges of evolutionary barriers, several different non-viral mRNA delivery systems have emerged, with liposomes and lipid nanoparticles (LNPs) being prominent and long-standing examples [94]. Liposomes are spherical vesicles with one (unilamellar) or more (multilamellar) phospholipid bilayers encapsulating an aqueous core, with polar head groups, DOTMA (1,2-di-O-octadecenyl-3-trimethylammonium-propane), and DOTAP (1,2-dioleoyl-3-trimethylammonium propane), and zwitterionic DOPE (dioleoylphosphatidylethanolamine). The realm of these lipid-based formulations has been witnessed in several transfection agents such as Lipofectamine for a successful mRNA delivery, both *in vitro* and *in vivo* [94, 95]. Nevertheless, the major caveat of using cationic liposomes following the induction of inflammation is elicited by the interferon-γ response in animal models (mice) [96]. Moreover, cationic liposomes such as DOTMA and DOTAP are prone to anionic serum protein neutralization, affecting their therapeutic potential with toxicity concerns. Lipid nanoparticles differ from liposomes by composition and their formulation scheme is comprehensively discussed elsewhere. In general, are composed of polyethyleneglycol (PEG) modified lipids, cholesterol, ionizable cationionic lipids and phospholipids [96]. Like liposomes, LNPs following endocytosis can translocate mRNA into endolysosomal compartments, where mRNA is degraded. In the subsequent intracellular trafficking events, LNPs are pumped out of the cell via exocytosis, which further impedes effective cellular delivery [96]. To circumvent these issues, ionizable lipids (amphiphilic–positively charged at low pH and neutral at pH 7) have been developed as illustrated in Figure 5 [95]. These ionizable lipid systems imparted endosomal escape functionality to internalizing nucleic acid-based drugs in a proposed phenomenon known as the proton sponge effect [97]. Although the exact mechanism is not known, it has been proposed that the positively charged LNP component facilitates interaction with the negatively charged endosomal membrane leading to ATP-driven acidification. These events follow a gradual shift in the pH from five to 6, rendering the endosomal membrane leaky by disrupting the membrane lipid bilayer. The details of this hypothesized phenomenon are reviewed comprehensively in previous reviews [97]. Resultingly, the nucleic acid cargo escapes the endosomal entrapment and is targeted toward its site of action. On the targeting front, further studies need to focus on developing RNA-based therapeutics using new targeting material and chemistries with enhanced endosomal escape properties [82, 95, 97].

Interestingly, one of the unique features of lipid nano formulations amenable to inefficient mRNA loading is a balanced ratio of ionizable lipids, and helper lipids such as 1,2-Dioleoyl-sn-glycero-3-PE (DOPE) and distearoylphosphatidylcholine (DSPC), which promote endosomal membrane fusion, polyethylene glycol (PEG) for improved bioavailability by reducing plasma protein binding, promoting cell-specific interactions toward non-hepatic organs, especially lymph nodes [98]. Besides translation outcomes of LNP-based mRNA delivery systems in protein replacement therapies, emerging mRNA-based vaccines are in the limelight for their role in cancer immunotherapies as rabies virus glycoprotein (RABV-G) mRNA-LNP based vaccines as shown in Figure 5. It is worth noting that the mRNA loading size in rabies virus LNP based vaccination varies from 1 to 15 µg per dose. For instance, previous studies elucidated sharp rise in humoral immune response in *in vivo* studies using range of dosages (0.1, 0.3, 3, and 10 µg) of RABV-G-mRNA; the high dose *in vivo* models of BALB/c mice with, 3 μg dose afforded maximum RABV-G-specific antibody titres (20.3 IU/mL, week 4) with single intramuscular (IM) injection [99–101]. In the subsequent immune response kinetic studies, it was revealed that a further 10 μg booster vaccination could increase and sustain long-term immune response kinetics with stable rise in Rabies virus neutralizing titres (1000–2000 IU/mL) in LNP formulation vs. empty LNPs (negative control) for up to 35 weeks [102]. Furthermore, lipid nanoformulation with helper lipids [e.g., cholesterol, dioleoylphosphatidylethanolamine (DOPE), dioleoylphosphocholine (DOPC), distearoylphosphatidylcholine (DSPC), and 1,2-Dimyristoyl-rac-glycerol (DMG-PEG) can further enhance the delivery efficacy by providing shielding effects and thus, stability against RNases and endonucleases within the cytosol and plasma membrane [103].

Thus, both lipoplexes and lipid nanoparticle-based mRNA formulations (Figure 5) and (Table 2) have already entered various stages of clinical trials for delivery toward extrahepatic tissues, seem promising, and have already broadened the horizon of RNA nanotechnology platforms.

### Cationic polymeric nanoparticles

**FIGURE 6:**
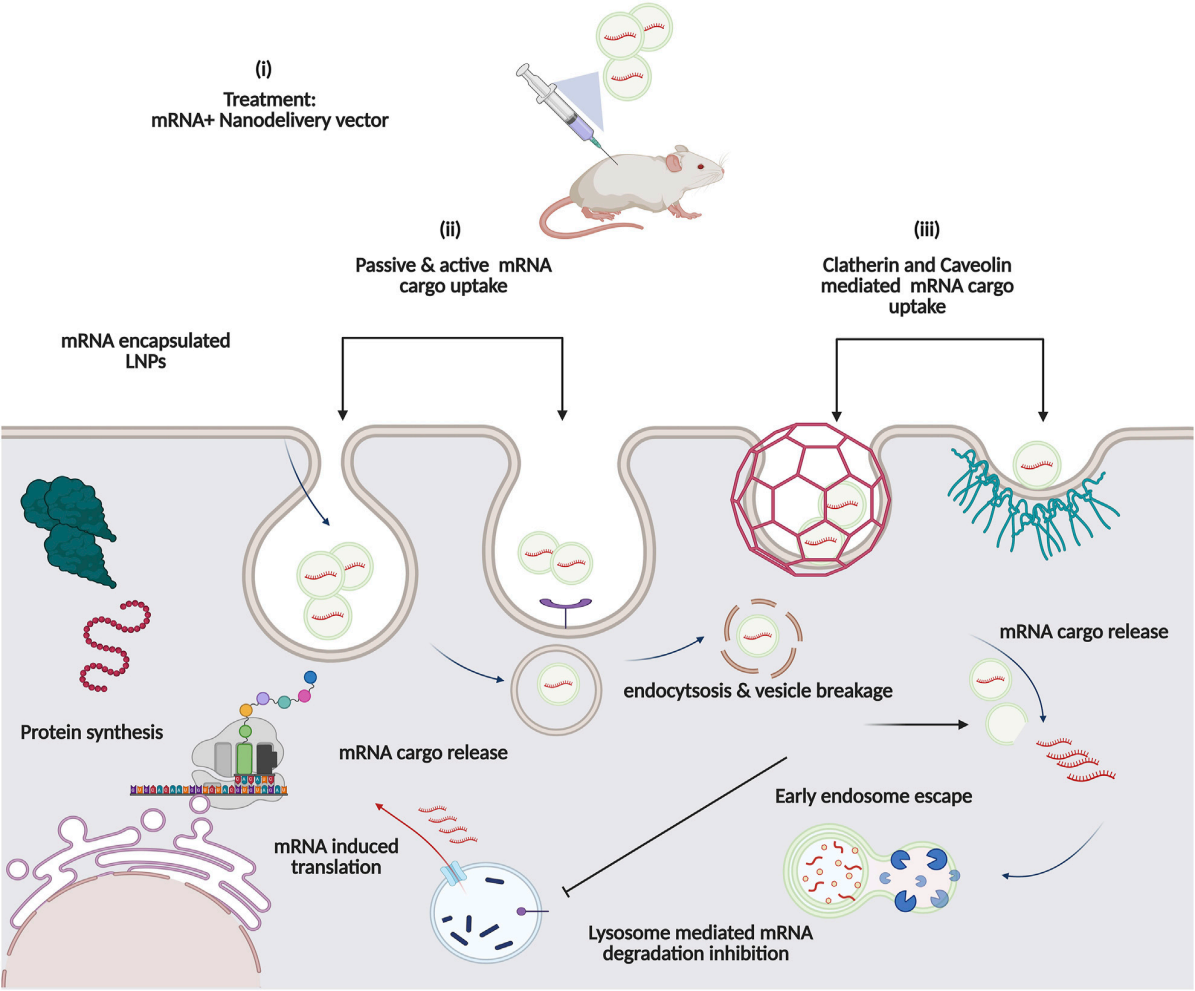
Proposed Intracellular pathway of Nanocarrier-based mRNA drugs: (i) *In vivo* immunisation with mRNA-based Nanovector formulation. Cell uptake of mRNA cargo occurs via either (ii) active (receptor-mediated) or passive diffusion across lipid bilayer or (iii) Clatherin and caveolin Mediated. Enhanced delivery with endolysosomal escape functionality aids in robust protein expression.

In the emerging era of nanoengineering, polymeric-based delivery vectors such as polymer-lipid hybrid nanoparticles, polymeric caged nanoparticles, dendrimer based lipid nanoparticles, etc., are some of the stand-out examples of non-viral RNA delivery systems, due to their inherent biocompatible and biodegradable nature [103]. In recent years poly(β-amino esters) (PBAE) has emerged in mRNA-targeted delivery to various parenchymal cells including pulmonary immune cells. For instance, OMPBAEs, an oligopeptide-modified PBAE, are designed for liver-specific targeting using mRNA transfection. Similarly, hyperbranched derivatives of PBAE maximized the mRNA delivery toward lung epithelium, shown in Figure 6 as polymer-caged mRNA nanovectors [104]. Moreover, in addition to terminal amino acids chemistries during formulation scheme, the mRNA loading efficiency and release kinetics is further influenced by polyplex size. For example, smaller sized (100–250 nm), biodegradable highly branched PBAE/mRNA complexes (Figure 5) showed high (80%) mRNA binding efficiency compared to larger (∼2 μm) counterparts [104]. In addition, to treat liver disorders, studies reported dendrimer-modified LNP systems used as promising theragnostics. For example, PEGylated BODIPY dyes to design PDB lipids for mRNA vaccine delivery to hepatic cells revealed therapeutic potential in liver cancers. Similarly, researchers used self-replicating and adjuvant-free mRNA vaccines against life-threatening infectious diseases (H1N1 influenza, Zika, Ebola, and Toxoplasma gondii) which made use of biodegradable polypropylene imine-based dendrimers or polyamidoamine (PAMAM-G1 mRNA vaccine) as shown in Figure 5 [105, 106].

Another category of intracellular nucleic acid delivery system is based on cell-penetrating peptides, which deliver mRNA cargo via various proposed mechanisms, i.e., either through lateral or passive diffusion penetrating the membrane barrier, involving clatherin or caveolin mediated cell uptake (Figure 6) [105, 106].

### Exosomes

Nevertheless, despite the emergence of these ascribed mRNA delivery systems, the poor stability and immunogenicity are still major drawbacks, seriously hampering their clinical utility. Moreover, due to the instability of naked mRNA, various protein replacement therapies have been regarded as an alternative strategy but cause highly immunogenic responses. The intrinsic properties of exosomes such as the “homing effect” make these biological nanocarriers an efficient and safe mRNA delivery vector [107]. First discovered in the 1980s, exosomes can transport proteins, nucleic acids (miRNAs and mRNAs), vitamins, and other intracellular cargo toward their target destination through signal transduction and cell-cell communication (Figure 5) [107, 108]. Previously, exosomes have been exploited to deliver mRNA toward immune cells to establish robust immunity against COVID-19 [107, 109]. These biologically derived extracellular vehicles (EVs) can target tumour cells by penetrating across the blood-brain barrier, making them an iconic mRNA-packing vector for brain targeting and other personalized therapies [110]. Yang explored *in vivo*, exosome-mRNA delivery which also enhanced survival rate with a significant reduction in tumour growth by targeting a tumour suppressor gene- Phosphatase and tensin homolog in *PTEN*-knockdown glioma mice [110].

Similarly, mRNA-encapsulated exosomes have also been explored as a promising collagen replacement therapy. For instance, exosomes derived from human dermal fibroblast were engineered with full length (4000 nt) long extracellular matrix alpha 1 collagen (COL1A1) mRNA (Figure 5). It was fascinating to discover that intradermal COL1A1-EV-mRNA delivery reduced dermal wrinkles in a photoaged UV irradiation mice model [111]. Additionally, inhalable EV-mRNA has shown robust delivery with higher efficiency compared to liposomes-mRNA delivery vectors [112].

Despite the outstanding characteristics of exosome-based mRNA delivery systems over LNPs, the challenges of using exosomes such as large-scale isolation and purification for subsequent clinical applications remain a paramount setback [107, 113]. The existing methods are not only costly but also generate low exosome yield. Furthermore, often using conventional methods like size-exclusion, immunoaffinity, ultrafiltration, etc. encounter contamination problems (with proteins or non-exosome vesicles) making the separation of therapeutically functional exosomes more tangible.

Immunogenicity and toxicity of exosomes have been the additional bottleneck in the design and optimization of efficient exosome-mRNA delivery systems [114]. Therefore, to have a must-have ‘mighty RNA delivery system,’ it is essential to adhere to GMP while selecting the most optimal and efficient exosome engineering method [115]. Understanding and determining vital cell sources for therapeutically functional exosomes is one of the first crucial steps to mitigate any contaminations and would potentially facilitate selective and efficient loading of mRNA cargo on these biologically active extracellular vesicles (Figure 5) [116]. In the future, it is anticipated that the choice of appropriate quality control measures together with standard protocols for production and characterization will be taken into consideration to generate reproducible and clinically functional exosomes [117].

### Hydrogels

In recent years, researchers have explored multifaceted role and drug release kinetics of variety of biomaterials loaded RNA [118, 119]. Hence, owing to good biocompatibility, and better biodegradability than LNP counterparts, hydrogels are one such biomaterial which have emerged as a promising injectable/implantable delivery platform for sustainable (burst/pulsating) RNA release. The tunable physiochemical features of hydrogels further enable the retention of functional mRNA activity in biomaterials-based immunotherapies [118, 120–122]. Hydrogels are three-dimensional porous and permeable scaffolds composed of either natural (chitosan/alginate), synthetic polymers, i.e., PEI, or even hybrid polymer complexes (hyaluronic acid and polyvinyl alcohol; HA.PVC) [119]. Furthermore, the robust design scheme of RNA-encased hydrogel platforms (Figure 5) depends on the nature of the loading cargo-whether a naked RNA cargo (siRNA, miRNA, mRNA) or additional nanocarriers (organic and inorganic nanoparticles, lipid or polymeric-based nano vectors) have been packaged onto hydrogel matrix. Thus, for effective localized delivery of naked RNAs with minimal off-target toxicities, the type of RNA-hydrogen interactions viz, covalent, electrostatic, hydrophobic, non-specific interactions or combination, play a significant role in regulating “on-demand” release profile, retention time, RNA stability with minimal off-target toxicities [118, 119]. A comparison of these conjugation strategies with technical limitations for efficient RNA packing is detailed comprehensively elsewhere [119]. Similarly, Yan et al. explored injectable chitosan/alginate hydrogel scaffolds (Figure 5) for rapid and controlled delivery of mRNA lipoplex (mRNA-OVA-A64 vaccine) *in vivo* (C578L/6J mice) [120]. Furthermore, in subsequent experiments, luciferase gene expression corresponding to IgG humoral response was found to be five-fold higher than the systematic injection [6]. Interestingly, stimuli response hydrogel-based RNA carriers (active or passive) have also been reported and engineered to modulate further the “on-demand/pulsating” RNA release profile, a crucial variable that affects downstream targeted RNA delivery approach. For instance, enzyme-responsive (matrix metallopeptidase MMP-2 degradable hydrogel), pH-responsive (DNA nanohydrogels for mRNA delivery), and Light sensitive hydrogels (UV-cleavable Chol-miR-26a) are among the proposed stimuli-responsive hydrogels utilized in “active” RNA cargo delivery, which were previously discussed [118, 119].

Rwandamuriye et al. have most recently engineered a hyaluronic acid-based hydrogel encapsulating an immunostimulant-a polyinosinic:polycytidylic acid poly(I:C) (a toll-like receptor 3-TLR3 agonist). Collectively, physiochemical characterizations including scanning electron microscopy, quantitative micro elastography, and Young’s Modulus confirmed the size, morphology as well as the surgically optimal hydrogel formulation (2.5% w/v HA and 3.5 mol% DTPH crosslinker) respectively, for safe and effective downstream biomedical applications. The resulting poly(I:C)-hydrogel optimized in surgical tumour model revealed promising outcomes in intraoperative immunotherapy, i.e., improved recurrence free-survival post-surgery, with transient alpha (IFNα) response rate and improved tumour sensitivity toward ICT tested mouse model (C57Bl/6J models). The safety and immunogenicity (type I IFN dependent responses) as well as the delivery efficacy of loaded immune adjuvants [poly(I:C)) in a proposed biomaterial matrix were further validated in canine veterinary models with cancer [121, 122]. The complete protocol guide on systematic “debulking surgery and bandaging method” of C57Bl/6J models followed by subcutaneous injection of poly(I:C) based hydrogels at tumour resection site are comprehensively explained in detail and published elsewhere [122].

Collectively, researchers proposed a robust alternative to neo-adjuvant and adjuvant immunotherapies which are only limited to certain cancer therapies, thus offering a facile advancement of exploiting low and prolonged RNA immunotherapeutic loaded hydrogels delivery vectors with broad spectrum biomedical applications from wound healing to anticancer and cardioprotective function against myocardial infraction.

## mRNA-based therapeutic strategies and applications

**FIGURE 7:**
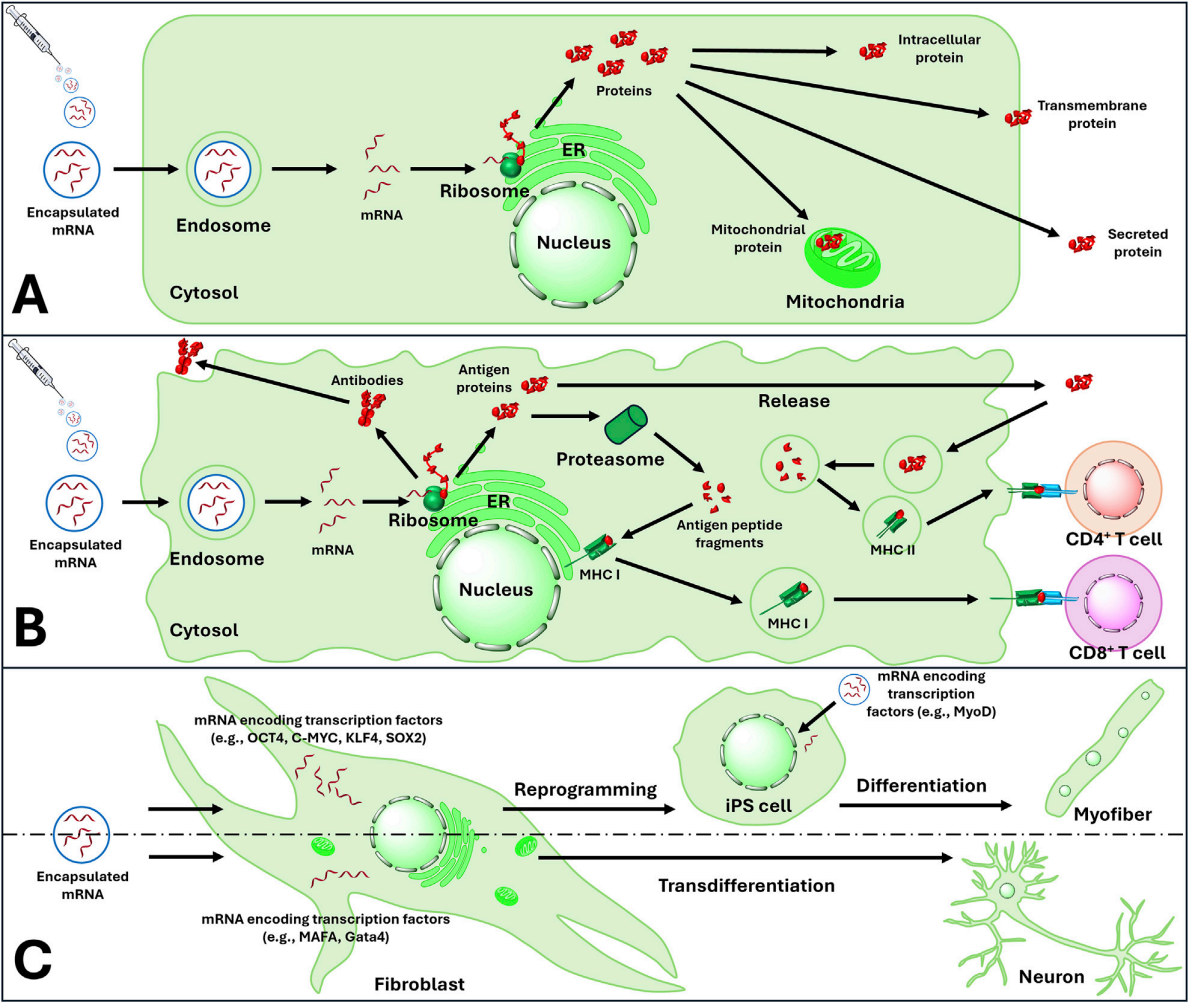
mRNA based therapeutic strategies include: **(A)** protein replacement therapy **(B)** Modulation of immunity by activating active and passive immunity and **(C)** reprogramming and transdifferentiation of audlt cells. ER, endoplasmic reticulum; MHC I, major histocompatibility complex class I; MHC II, major histocompatibility complex class II; CD4, cluster of differentiation 4; CD8, cluster of differentiation 8; iPS cells, induced pluripotent stem cells.

In general, eukaryotic mRNA translation initiation can be divided into three stages. Firstly, 40S ribosome subunit binds to mRNA in the vicinity of 5′Cap with the assistance of several eukaryotic initiation factors (elFs). Subsequently, the 40S ribosome scans through the 5′UTR to find the initiation codon, and eventually, 60S ribosomal subunit joins the 40S subunit releasing the elFs. As a result, 80S ribosome is formed and starts the process of elongation [52]. Unlike *in vivo* synthesised mRNA, synthetic mRNA enters the cell from extracellular space; yet both display their activity in cytoplasm without integrating to the genomic DNA (Table 1). Using host machinery, IVT mRNA is translated into peptide which undergoes post-transcriptional modification to become bioactive protein. IVT mRNA is designed to resemble the natural post-modified mRNA in the cytoplasm [123]. mRNA-based therapeutics can be divided into three main therapeutic strategies: cellular modulation, protein replacement, and immunomodulation. The cellular modulation and protein replacement therapeutic strategies involve mRNAs that encode a functional protein, which can be expressed in the target cell as an endogenous protein, a functional foreign protein or proteins used for gene editing or cellular modulation (Figure 7; Tables 1, 2). In the context of mRNA-based immunomodulation, the introduced mRNA expresses antigens that trigger immune responses, either cell-mediated or antibody-mediated, against an external pathogen or a tumour cell [104].

**TABLE 1:** mRNA therapeutic Strategies.

Therapeutic strategies	Applications	References
mRNA based protein replacement	Cardiac, collagen replacement, treatments for rare genetic disorder	[12, 17, 104, 111]
mRNA based immunomodulation	mRNA encoded monoclonal antibody therapy	[104, 125, 126]
	mRNA- based CD4^+^ T cell therapeutics	[104, 127]
	mRNA-based T lymphocyte therapeutics (editing primary T cells)	[17, 104, 128]
mRNA based cellular modulation	Induced pluripotent stem (iPS) therapeutics Gene editing	[17, 68, 104, 129]

**TABLE 2:** mRNA therapeutic development.

Therapeutic strategy	Disease or condition	NCT no.	Candidate name	Molecular target	Delivery vehicle	Administering method	Sponsor	Development stage	References
mRNA based immunomodulation	COVID-19	NCT04860297	mRNA-1273	S-2P	LNP	Intramuscular injection	ModernaTX, Inc	III	[205]
		NCT04816669	BNT162b2	S-2P	LNP	Intramuscular injection	BioNTech SE	III EUA & CMA	[206]
		NCT04523571	BNT162b1	Trimerized RBD	LNP	Intramuscular injection	BioNTech SE	I	[207, 208]
		NCT04847102	ARCoV	RBD	LNP	Intramuscular injection	Walvax Biotechnology Co., Ltd.	III	[209]
		NCT04668339	ARCT-021	Naive S protein	LNP/	Intramuscular injection	Arcturus Therapeutics, Inc.	II	[210]
		NCT04798027	MRT5500	S-2P with a furin cleavagesite mutant	LNP	Intramuscular injection	Sanofi Pasteur, a Sanofi Company	I/II	[211]
		NCT04566276	ChulaCov19	Transmembrane S protein	LNP	Intramuscular injection	Chulalongkorn University	I/II	[212, 213]
	Rabies	NCT02241135	CV7201	Rabies virus glycoprotein (RBVG protein)	Protamine-condensed mRNA	Intradermal injection	CureVac	I	[214, 215]
		NCT03713086	CV7202	RBVG protein	LNP	Intramuscular	CureVac	I	[216, 217]
	Influenza	NCT04956575	mRNA-1010	Hemagglutinin (HA) from 4 influenza virus strains (influenza A (H1N1) Influenza A (H3N2) Influenza B (Yamagata lineage) Influenza B (Victoria lineage))	LNP	Intramuscular injection	ModernaTX, Inc.	I/II	[218]
		NCT05333289	mRNA-1020 and mRNA-1030	Haemagglutinin and neuraminidase to conserved regions of the virus	LNP	Intramuscular injection	ModernaTX, Inc.	I/II	[219, 220]
		NCT06431607	GSK4382276A	HA from influenza virus antigens (H1N1, H3N2, Yamagata lineage, Victoria lineage)	LNP	Intramuscular injection	GlaxoSmithKline	II	[221]
		NCT05823974			LNP	Intramuscular injection	GlaxoSmithKline	I/II	[222]
	Chikungunya virus infection	NCT03829384	mRNA-1944	Anti-Chikungunya antibody	LNP	Intravenous infusion	ModernaTX, Inc.	I	[223]
	HIV infection	NCT05217641	HVTN 302	Stabilized HIV envelope (Env) trimers Native-like HIV-1 Env trimers (BG505 MD39.3, BG505 MD39.3 gp151 and BG505 MD39.3 gp151 CD4KO)	LNP	Intramuscular injection	National Institute of Allergy and Infectious Diseases (NIAID)	i	[224]
		NCT05001373	IAVI G002	eOD-GT8 60mer Env (mRNA-1644) Core-g28v2 60mer Env (mRNA-1644v2)	LNP	Intramuscular injection	International AIDS vaccine initiative	I	[225]
		NCT05903339	HIV-1 ferritin nanoparticle vaccine	Native-like HIV-1 envelope trimer	LNP	Intramuscular injection	National Institute of Allergy and Infectious Diseases (NIAID)	I	[226]
	Cytomegalovirus infection	NCT03382405	mRNA-1647/1443	CMV glycoprotein H (gH) pentamer complex	LNP	Intradermal	ModernaTX, Inc.	I	[227–229]
	Herpes Zoster (HZ)	NCT05701800	mRNA-1468	Glycoprotein E	_	Intramuscular	ModernaTX, Inc.	I/II	[230]
		NCT05703607	BNT167	Glycoprotein E	_	Intramuscular	Pfizer	II	[231]
	Human metapneumovirus and parainfluenza infection	NCT03392389	mRNA-1653	Human metapneumovirus and human parainfluenza virus type 3 vaccine	Lipid nanocarrier	Intradermal	Moderna	I	[232, 233]
	Melanoma	NCT01676779	TriMixDC-MEL	MAGE- A3, MAGE-C2, tyrosinase and gp100	DCs	Intradermal injection	Universitair Ziekenhuis Brussel	I	[234, 235]
		NCT04526899	BNT111	NY-ESO-1,tyrosine,MAGE-A3, and TPTE)	Lipoplex	Intravenous	BioNTech	II	[236, 237]
	Triple negative breast cancer	NCT02316457		[1] 3 TAAs selected from a warehouse and p53 RNA; [2] Neo-Ag based on NGS screening	Lipo-MERIT, DOTMA(DOTAP)/DOPE lipoplex	Intravascular	BioNTech	I	[238]
	NSCLC	NCT03164772	CV9202	NY-ESO-1, MAGE-C1, MAGE-C2, survivin, 5T4, and MUC1	Protamine-condensed mRNA	Intradermal injection	Ludwig institute for cancer research	I/II	[239, 240]
		NCT03948763	-	K-ras encoding mutation (mRNA-5671)	Lipid nanocarrier	Intramuscular	Merck sharp &Dohme	I	[241, 242]
	Colorectal cancer	NCT04486378	RO7198457	Personalised neoantigen	Lipid nanocarrier	Intravascular	BioNTech	II	[243]
	Head and neck cancer	NCT04534205	BNT113	E6 & E7 oncoproteins of HPV16	Lipoplex	Intravenous	BioNTech SE	II	[244–246]
	Solid Tumours	NCT04503278	BNT211-01	Claudin 6 (CLDN6) chimeric antigen receptor T cells (CAR-T)	Lipoplex	Intravenous	BioNtech & gene Therapies	I	[247, 248]
		NCT03313778	mRNA-4157	Neo- Ag	Lipid nanocarrier	Intradermal	Moderna	I	[249–251]
	High-risk melanoma	NCT05933577	mRNA-4157 (V940) with pembrolizumab MK-3475				Merck sharp & Dohme LLC.	III	[252]
	Non-small cell lung cancer	NCT06077760					Merck sharp & Dohme LLC.	III	[253]
	Pancreatic cancer	NCT04161755	_	Personalise neo-antigen (neo-Ag)	Lipoplex	Intravenous	Memorial Sloan Kettering Cancer Centre, USA	I	[254, 255]
	Autologous cancer	NCT03480152	mRNA 4650	Neo-Ag mRNA	LNP	Intramuscular	National Cancer Institute (NCI)	I/II	[256, 257]
	Methylmalonic acidaemia	NCT03810690	mRNA-3704	Methylmalonyl-CoA mutase	Unknown LNP	Intravenous injection	ModernaTX, Inc.	I/II (Withdrawn)	[258]
mRNA based protein replacement therapy	Propionic acidaemia	NCT04159103	mRNA-3927	Propionyl-CoA carboxylase	Unknown LNP	Intravenous injection	ModernaTX, Inc.	I/II	[259]
	Ornithine transcarbamylase deficiency (OTD)	NCT05526066	ARCT-810	Ornithine transcarbamylase	LNP	Intravenous injection	Arcturus Therapeutics, Inc.	II (ongoing)	[260, 261]
	Cystic fibrosis	NCT05668741	VX-522	CFTR	LNP	Inhalation	Vertex pharmaceuticals incorporated	I/II	[262, 263]
		NCT03375047	MRT5005	CFTCR	LNP	Inhalation	Translate Bio, Inc	I/II	[264, 265]
	Type II diabetes	NCT02935712	AZD8601	VEGFA	Nake mRNA	Intradermal injection	AstraZeneca	I (completed)	[266, 267]
	Heart failure	NCT03370887	AZD8601 EPICCURE	VEGFA	Naked mRNA	Epicardial injection	AstraZeneca	II (completed)	[268–270]
mRNA based cellular modulation	Transthyretin amyloidosis with polyneuropathy (TAP)	NCT04601051	NTLA-2001	CRISPR–Cas9	LNP	Intravenous injection	Intellia Therapeutics	I	[271, 272]
		NCT06128629						III	[273]
	Hereditary transthyretin amyloidosis	NCT04601051	NTLA-2001	CRISPR-Cas9–based transthyretin (TTR) editing	Lipid nanocarrier	Intravascular	Intellia Therapeutics	I	[272, 274]

Since mRNA’s discovery, researchers have envisioned its therapeutic potential. SARS-CoV-2 prompted extensive resources for mRNA-based treatments and vaccines, yielding technological breakthroughs. These advances reveal synthetic mRNA’s therapeutic possibilities, warranting thorough patent examination [124].

### mRNA-based protein replacement

mRNA based protein replacement therapy (PRT) is defined as treatment that increases the concentration of a specific protein that has been deficient in a cell due to absent or incomplete synthesis because of a genetic mutation. mRNA possesses the capacity to encode the protein that is deficient in the patient [12]. Therefore, mRNA-based protein replacement has less risks in mutagenesis. mRNA designed and synthesised with protein coding region of the target gene incorporating modified nucleotides, has shown to synthesize proteins in mouse models with low immunogenicity, prolonged stability and high efficiency [17]. Following these principal examples, we explore the innovative patented therapies applying IVT mRNA as protein replacement and cellular modulation therapy.

#### Citrullinemia type 2 (OMIM ID 603471; 603859)

Citrullinemia type 2 (CTLN2) or adult-onset type 2 citrullinemia, is a class of urea cycle disorders, inherited as an autosomal recessive trait [130]. CTLN2 is characterized by markedly high levels of L-citrulline in both the liver and blood of affected patients with a severe Citrin deficiency (CD) [130–134]. Epidemiological studies have shown that prevalence rates for pathogenic bi-allelic variants are commonly found in East Asian populations; mainly Japan with an incidence rate of 1:69 and 1:65 in China [135, 136]. The occurrence rate of CTLN2-induced Citrin deficiency was most recently reported in France, UK and Canada, which implies that CD has now emerged as a pan-ethnic disease worldwide [135, 136].

The genetic studies elucidated that the underlying cause of Citrin deficiency is due to the mutation in SLC25A13-a mitochondrial localized liver-specific aspartateS/glutamate transporter encoding gene for citrin protein, located on 7q21.3 locus [133, 136, 137]. The citrin/aspartate/glutamate carrier isoform 2 (AGC2) plays a critical role in hepatocyte glycolysis by providing a mitochondria-specific energy shuttle in the form of Ca^+^ dependent malate-aspartate nicotinamide adenine dinucleotide hydrogen (NADH) carrier [131, 135]. In citrin-deficient individuals, without NADH, ammonia is unable to be removed during the urea cycle leading to symptoms of hyperammonia [135]. The metabolic pathways involving *de novo* lipogenesis, fatty liver-specifically non-alcoholic fatty liver disease (NAFLD) and hyperlipidemia have been well documented in scientific literature [131, 132, 135, 136, 138].

Given the severity of the disease manifestation in CD patients with severe hyperammonemia conditions, liver transplantation is generally the last resort to manage the clinical pathology of CTLN2 [132, 139–142]. Presently, recommended dietary therapies for adult-onset type II citrin deficiency are mostly main chain triacylglycerol (MCT) oils (5 mL twice daily), Sodium pyruvate (0.1–0.3 g/kg/day), low carbohydrates and high protein supplements [132, 133, 140, 142]. In practice, patient compliance, recorded poor efficacy and reported side effects (carbohydrate toxicity) have lessened the effectiveness of traditional treatment methods [131, 132, 134, 139, 142]. Treatment regimens mentioned earlier has succeeded in the complete management of inherited metabolic CTLN2 disorder [131, 136, 139].

In 2019, Cao developed a safe and effective mRNA therapy based on LNPs, bearing a codon optimised and chemically modified mRNA variants for human citrin (citrin-mRNAcov) [134]. Subsequent intravenous (IV) administration to both mammalian liver cell-lines (HepG2) *in vitro* and CTLN2 mouse model *in vivo* corrected the subcellular localisation and expression of a fully functional citrin protein in liver mitochondria, i.e., almost equivalent to 2–5% of wild-type expression levels [134]. Accordingly, in 2022, Martini (US patent NO. 20220071915A1) designed intracellular mRNA-lipid nanoparticle-based delivery technology encoding a full functional fragment of citrin protein, sharing 85% sequence homology to wild-type human Citrin Isoform 2 (Seq ID No. 3) [136]. Furthermore, the designed mRNA-based LNP technology further addressed the hepatic citrin levels (increased by 50%), a key biomarker for fatty liver in citrullinemia type 2 patients. The hepatic citrin remained at optimal levels for 24–96 h post-administration [136].

#### Acute intermittent porphyria (OMIM ID 176000)

Acute intermittent porphyria (AIP, also known as Swedish porphyria) is a rare metabolic disease that is characterized by deficient heme biosynthesis and inherited in autosomal dominant manner [143, 144]. AIP occurs because of mutations in the gene encoding porphobilinogen deaminase (PBGD), also known as hydroxymethylbilane synthase (HMBS). The morbidity of AIP is 1 in 20,000 [145], and more than 200 pathogenic mutations have been recognized [143]. Defects of PBGD lead to insufficient heme biosynthesis, which stimulates upregulation of *δ-aminolevulinic acid* synthase (ALAS) and eventually accumulates neurotoxic metabolic intermediates ALA and PBG [146–150]. Intravenous heme administration is a useful treatment for acute attacks, as it suppresses the physiological feedback of insufficient heme storage, thus reducing the production of ALA and PBG, because of ALAS inhibition. Liver transplantation is the definitive solution for AIP patients [151–153]. mRNA encoding PBGD for the treatment of AIP has been patented [154], and this strategy not only restores the expression of PBGD and its activity but also decreases the level of the neurotoxic metabolites (ALA and PBG). Notably, intravenous administration of 0.5 mg/kg PBGD mRNA formulated by lipid nanoparticles into Cynomolgus macaque achieved ∼40% and ∼90% increases in the activity and expression level of PBGD, respectively, in comparison to the phosphate buffered solution (PBS) treated animals [154].

#### Argininosuccinate synthetase deficiency (OMIM ID 215700)

Arginosuccinate synthetase deficiency (ASD) also known as argininosuccinic acid synthase deficiency and citrullinemia type I, is a urea cycle disorder (UCD). In UCDs, ammonia (nitrogenous waste of protein metabolism) fails to be converted into urea and released through urine. The resultant accumulation of ammonia in the body causes toxic effects. Arginosuccinate synthetase is a key enzyme in urea cycle, and deficiency of this enzyme leads to inability to catalyze the formation of argininosuccinic acid. This process results in the buildup of upstream substrates, specifically citrulline and aspartic acid, which eventually leads to UCD and hyperammonemia. ASD is inherited in an autosomal recessive pattern and is attributed to mutations in the ASS1 gene responsible for encoding arginosuccinate synthetase [155, 156]. The prevalence of ASD is ∼1/57,000 [157], and for patients with ASD, nutritional management is essential in preventing complications of hyperammonemia [158]. An mRNA therapy for ASD has been patented [159], providing an mRNA encoding arginosuccinate synthetase that is able to restore sustained and highly efficient *in vivo* synthesis of the enzyme, when delivered in lipid nanoparticles. Furthermore, administration of the mRNA (1.0 mg/kg) to *ASS1* knockout mice achieved ∼50% reduction of ammonia in plasma on the following day [159].

#### Cystic fibrosis (OMIM ID 219700)

Cystic fibrosis (CF) is an autosomal recessive genetic disorder caused by pathogenic variants of the gene encoding cystic fibrosis transmembrane conductance regulator (CFTR) [160–162]. CFTR is a transmembrane protein that forms a chloride channel. Mutations in the *CFTR* gene lead to synthesis of defective proteins that are unable to carry out chloride ion transport or even cannot reach the cell membrane. The resultant accumulation of chloride ions leads to generation of thick secretions (mucus) in different organs such as lungs and liver [163]. Patients with CF eventually suffer from loss of lung function as a result of progressive lung disease [164, 165]. Currently there are ∼89,000 people affected by CF worldwide [165]. First-line treatments for the CF-associated lung disease consist of administration of mucolytics (mucus thinner), anti-inflammatories, airway clearance, and antibiotics. Small molecular CFTR modulators such as ivacaftor have been approved by regulatory bodies as well [165]. An mRNA treatment for CF has been patented [166], providing an *CFTR* mRNA encapsulated in lipid nanoparticles that can increase the expression level and/or activity of CFTR in subjects, thus reducing the production of toxic metabolites associated with CFTR deficiency and/or dysfunction [166].

#### Fabry disease (OMIM ID 301500)

Fabry disease is a progressive, X-linked inherited lysosomal storage disorder (LSD), characterized by deficient activity of lysosomal α-galactosidase A (α-Gal A), affecting glycosphingolipid metabolism [167–170]. α-Gal A is coded by *GLA* gene, and to date over 1,000 mutations have been identified in the gene, most of which lead to synthesis of a non-functional protein [170, 171]. The incidence of Fabry disease is reported as 1 in 117,000 [172], however, the data may underestimate the exact disease frequency due to the rarity of the disease and undiagnosed patients [169, 170]. α-Gal A cleaves the terminal galactose from globotriaosylceramide (Gb3), deficiency of α-Gal A leads to progressive accumulation of Gb3 and its analogue lyso-Gb3 (globotriaosylsphingosine) within lysosomes in most cell types, thus affecting different tissues and organs [173,174]. Over time, lysosomal storage of these glycosphingolipids causes cellular dysfunction and damage, eventually leading to organ failure such as cardiovascular complications and end-stage renal disease [175, 176]. Although associated with side effects, enzyme replacement therapy (ERT) is the current standard of care for Fabry disease, with Replagal® and Fabrazyme® commercially available [177–179]. Another approach is chaperone therapy, as chaperones bind to defective α-Gal A and help with its functioning. However, a limitation of this therapy is that only the patients with amenable mutations can be treated [169]. Martini’s group reports the employment of mRNA coding α-Gal A towards treating Fabry disease. Notably, a single intravenous administration (0.5 mg/kg) of α-Gal A mRNA into Fabry mice achieved long-lasting (at least 12 weeks) attenuation of Gb3 accumulation in tissues, suggesting that less frequent dosing of mRNA therapy may be required compared to the conventional ERT therapy, which is administered biweekly [180].

#### Phenylketonuria (OMIM ID 261600)

Phenylketonuria (PKU), also known as phenylalanine hydroxylase (PAH) deficiency or Følling disease, is an autosomal recessive inborn error of phenylalanine (Phe) metabolism [181]. PKU is the most common metabolic defect of an amino acid in humans, with an average prevalence worldwide of ∼1:10,000 live births [182]. PAH is a hepatic enzyme responsible for converting Phe to tyrosine (Tyr) through Phe hydroxylation [181, 183]. PKU is caused predominantly by pathogenetic mutations in the *PAH* gene, and more than 1,000 such variants have been identified [184]. PAH deficiency results in accumulation of Phe in the body, leading to hyperphenylalaninaemia (HPA). Spontaneous deamination of Phe results in the formation of phenylpyruvate and other phenylketones, which are excreted in urine [182]. The toxic effect of HPA causes brain dysfunction, and if left untreated, PKU patients develop intellectual disability. Dietary restriction of Phe is the prevailing treatment, preventing intellectual disability in early treated patients. However, issues associated with medical foods include poor compliance (due to palatability of the diet) and nutritional deficiencies [185–187]. mRNA therapy is a promising alternative treatment for PKU. Notably, biweekly administration of *PAH* mRNA (1.0 mg/kg) encapsulated within lipid nanoparticles significantly reduced the level of serum Phe (from more than 1500 μM to less than 500 μM) in PAH knockout mice [188].

#### Pope disease (OMIM ID 232300)

Pompe disease (PD), the first documented LSD, is also known as glycogenosis type II, type II glycogen storage disease (GSDII), or acid maltase deficiency [189–192]. PD is inherited in an autosomal recessive manner, and is a chronic and severe metabolic myopathy caused by mutations in the gene encoding acid α-glucosidase (GAA) [189–191, 193, 194]. The estimated prevalence of PD, dependent on different ethnic groups and geographic regions, is ∼1–9 in 100,000 newborns [195–197]. In lysosomes, GAA degrades glycogen into glucoses in acidic pH by hydrolyzing glycogen’s α-1,4 and α-1,6-glucosidic linkages [198–200]. Partial or total GAA deficiency (due to mutations) induces lysosomal accumulation and storage of glycogen in cells of multiple tissues, leading to lysosomal rupture, cell malfunctions and autophagy [190, 192, 201]. Cardiac and skeletal muscles are the most affected tissues. Currently, ERT using recombinant human GAA (rhGAA) is the only clinically approved treatment for PD, improving the respiratory and cardiac functions of patients and increasing their life expectancy [202]. However, immunologic reactions against rhGAA and poor delivery and uptake of the enzyme to muscles limit the efficacy of ERT [191, 203]. An mRNA therapy for PD has been patented [204], providing a *GAA* mRNA encapsulated in lipid nanoparticles that can restore efficient and sustained enzyme expression *in vivo*, thus reducing the storage of glycogen in liver and muscle cells.

### mRNA based cellular modulation

mRNA based cellular modulation is used in regenerative therapies to supplement defective tissue with molecular compounds necessary for restoring them to their functional physiological state. This restoration process requires a variety of proteins like growth factors, cytokines, and transcription factors that are essential for cell division and growth. You et al. successfully produced EVs by cellular nanoporation of human dermal fibroblasts and encapsulation of exogenous mRNA encoded with extracellular-matrix α1 type-I collagen (COL1A1). The EVs loaded with *COL1A1* mRNA was intradermally delivered to mice with collagen depleted dermal tissue via microneedle arrays. The corresponding *COL1A1* mRNA protein was identified starting from 12 h post and peaked at 4 days after delivery, successfully reducing the wrinkles in photoaged skin [111]. It is evident that mRNA can be potentially encoded with proteins that could modulate the cellular characterises. Yamanaka et al. designed mRNA encoding transcription factors such as OCT3/4, SOX2, MYC and KLF4 (currently known as Yamanaka factors). Transfecting these transcription factor-encoded mRNAs to somatic cells reprogramed them into induced pluripotent stem cells (iPSC) [275].

Sun et al. investigated the potential of using modified mRNA (AZD8601) encoded with vascular endothelial growth factor A (VEGF-A) as a therapeutic intervention for diabetic wound healing. The study displayed accelerated dose dependent neo-vessel formation, re-epithelialization and blood flow upregulation, facilitating tissue regeneration [276]. Above results were confirmed by the study conducted by Zha et al. where they delivered VEGF-A mRNA via LNP for wound healing in diabetic induced mice [277]. Recently, Antila et al. have been exploring the therapeutic potential of AZD8601 in regeneration of cardiac muscles after bypass surgery, however, they were unable to find a significant difference in efficacy in regeneration compared to the placebo in human trial even though promising results were observed in animal trials. Further studies are ongoing to improve the therapeutic efficacy and delivery [268, 269, 278].

### mRNA-based immunomodulation

Basic human immunity can be divided into two main categories: active and passive immunity. This study reviews the utilization of synthetic mRNA to exploit the active and passive immune pathways in the human body as functional anti-viral and anti-cancer therapies (Figure 7). Pardi et al. developed the world’s first mRNA-based platform for delivering the heavy and light chains of the anti-HIV-1 antibody (VRC01) and demonstrated that mice transfected from mRNA encoded with anti-HIV-1 antibody (VRC01) produces VRCO1 antibodies [279, 280]. These findings highlight the feasibility of using encoded mRNA for both active and passive immune pathways for therapeutic intervention, laying the foundation of numerous innovations displayed below.

### Anti-viral therapies

#### Human cytomegalovirus infections

Human cytomegalovirus (HHV-5) is a herpesvirus from the Herpesviridae family, transmitted via infected bodily fluids [281, 282]. Shwartz et al. conducted single-cell transcriptomic studies to elucidate the molecular pathogenesis in infected individuals involving HMCV latency and reactivation [281]. Previous studies identified glycoprotein subunit gB as a significant mediator of viral entry in fibroblast and epithelial cells [283, 284]. Epithelial and endothelial cells are often infected due to their expression of receptors that facilitate the entry of the pentameric complex comprising gH/gL/UL128-131 glycoproteins [285, 286].

Epidemiological data indicate that CMV poses a considerable global health challenge, affecting individuals of all ages [287]. Furthermore, among all live-born infants, the incidence of congenital cytomegalovirus (cCMV) infection is 1 in 200 in high-income countries and 1 in 71 in low-income countries [288].

Treating human cytomegalovirus (hCMV) is challenging, with FDA-approved drugs like cidofovir and ganciclovir contraindicated due to cross-resistance and toxicity. Current investigational hCMV vaccines, including mouse cytomegalovirus (mCMV) vaccine targeting viral G-protein-coupled receptors (M33), avoid systematic CMV spread only in animal models [285]. The gB/MF59 vaccine failed to block viral entry to epithelial cells [284]. Kschonsak (2022) revealed the structural basis for hCMV epitope engagement involving pentamer-thrombomodulin complex and viral entry mechanism [289]. Antibodies blocking viral entry have lower potency to neutralize glycoprotein B than those recognizing quarternary neutralising epitopes formed by pentameric complex-gH/gL/UL128/UL130/UL131 [284].

In 2023, Ciaramella (2023) (Moderna Inc., USA.) invented an mRNA-based LNP vaccine formulation (encoding pentameric complex +gB glycoprotein subunit plus compound 25: cholesterol) against hCMV [286]. This mRNA-based hCMV vaccine showed safe and effective immunogenic responses to prevent the maternal-fetal transmission of congenital viral diseases, especially herpes virus (HSV-5) [290]. The design rationale of this broad-spectrum mRNA vaccine against hCMV was to express at least one polypeptide encoding hCMV(gH, UL130, 131, UL 128) and one polypeptide expressing hCMV gB, both co-expressed within a non-viral delivery construct-a lipid-based nanoparticle formulation [286]. In Phase 1 trail, following both priming (4.2 µg) and boosting doses (1.4 µg) in *in vivo* models (balb/c mice model), this second generation hCMV mRNA vaccines encoding the pentamer and gB formulated with Compound 25 lipids elicited strong pentamer-specific antibody titres above 3 log10 at two different time points (day 40, 3 weeks post prime; day 40, 3 weeks post boost), measured by ELISA. Further experimental data highlighted the significance of using CytoGam (a hyperimmune serum for HCMV prophylaxis) as a benchmark to compare the neutralising HCMV antibody titers. Similarly, Moderna’s CMvictory phase 3 trial NCT05085366 is currently investigating safety, efficacy and immunogenicity of LNP-mRNA 1647 (multi-antigenic vaccine encoding pentameric variant: gL-UL128-UL130-UL131A and a glycoprotein complex) in healthy adults against CMV infection [291]. With robust long-lasting T and B cell responses, the Phase 3 trial was concluded with the promising therapeutic potential of mRNA-1647 vaccine and early prevention of CMV infection [286]. This implies that current investigational vaccines at various stages of clinical trial involving nano-engineered RNA technology and LNP formulations serve as an iconic prophylactic strategy. From ongoing clinical studies, they are found to be effective in the prevention and spread of congenital cytomegalovirus infection in high-risk groups (from pregnant mothers to unborn fetuses, infant patients and Allogenic hematopoietic cell transplanted patients) [286, 292].

#### Betacoronavirus

Betacoronaviruses (Beta CoVs), from the Coronavirinae subfamily, are pathogenic respiratory viruses that have severely impacted global populations [293, 294]. They are (30 kb) positive-sense, enveloped, single-stranded ribonucleic acid (RNA) viruses further comprising 4 lineages (A, B, C and D) [293]. SARS-CoV-2, an emerging Beta CoV, raised significant health and economic concerns [293, 294]. Beta lineage has affected over 90 countries [295]. Unlike other Beta CoVs such as severe acute respiratory syndrome (SARS) and Middle East respiratory syndrome (MERS), patients with COVID-19 are mostly asymptomatic [296]. Yet SARS-COV-2 infections are more life-threatening due to high contagiousness [294, 297]. Human-human transmission drives elevated cases, with bats and pangolins as potential intermediate vectors [293, 294].

Females are less susceptible to Beta CoVs due to higher biallelic X-linked immune-related gene expressions involving TLR-7 and cytotoxic T cell responses [293]. Sharma and colleagues have further shown that angiotensin-converting enzyme 2 (ACE 2) – a key viral contact and entry point for spike glycoproteins of SARS-CoV, is highly expressed in testis compared to ovaries [298]. During infection, these viruses elevate interleukins (IL-6 and IL-8) suggesting estrogen plays a stimulatory role in the sex-specific innate and adaptive immune responses [293].

Given the sex-specific differences in immunological reactions to both vaccination and COVID-19 infection, several sex hormonal therapies entered pre-clinical and clinical studies to treat both men and women using specific medical interventions [293, 299], for example, anti-androgen treatment (Clinical ID: NCT04446429) and progesterone for the treatment of COVID-19 in hospitalized men (Clinical ID: NCT04365127), both completed in 2021 [293]. Since the outbreak, various biomedical and pharmacotherapies entered the pipeline, among them are COVID-19 antiviral drugs of various classifications; such as remdesivir (nucleotide analogue), tocilizumab (biologics), various RNA synthesis inhibitors, and protease inhibitors like ritonavir/lopinavir combination therapies, etc [293, 299].

Understanding molecular pathogenesis and sex-specific immune responses has led to lipid-based nanoformulations with mRNA sequences against betacoronaviruses [294]. In 2020, Ciaramella and Himansu (Moderna Inc.) developed recombinant mRNA vaccines containing ORF encoding Spike glycoprotein [294]. This patented mRNA vaccine comprises of 2 constructs First, mRNA encoding coronavirus spike glycoprotein receptor binding domains. Second mRNA construct encoding proinflammatory cytokines (Interleukins, TGFs, interferons, TNF-α, M-CSFs, and GM-CSF) [294].

Today, only two mRNA-based vaccines against beta CoVs have received full US Food and Drug (FDA) approval, notably the Pfizer-BioNTech and Moderna COVID-19 vaccine [293]. By 2021, the unprecedented 5.42 million deaths due to COVID-19 and the red alert established by the WHO has reinforced the urgency of establishing an effective and safe non-viral RNA delivery platform [300, 301].

#### Lassa virus mRNA vaccine

The Lassa mammarena virus (LASV) from Arenaviridae is one of the most lethal viral haemorrhagic fever (VHF) viruses. VHF viruses can invade vascular organs causing cardiovascular dysfunction and hepatitis [302] LASV infection is endemic in Western Africa with 5,000 deaths yearly, with cases reported in the UK, Netherlands and Germany [302, 303]. Due to increasing seroprevalence and LASV lineages (I-VII), they are on WHO’s high-priority list [304].

LASV has an 80–200 nm enveloped RNA genome encoding four proteins: nucleoprotein (NP), glycoproteins complex (GPC), zinc-binding domain and large proteins. GPC enables viral attachment and entry through endosomal pathways [302]. A glycan shield around GPC helps evade host immune responses through epitope switching, blocking LASV-specific antibody neutralization [302, 305].

Currently, to treat fatal Lassa fever, many small molecules have entered clinical trials, such as favipiravir and ribavirin combination therapy and a novel LHF-535 (the enhanced analogue of benzimidazole derivative), which is a viral entry blocker [302, 306]. While other immunotherapies such as Arevirumab-3 succeeded in providing complete immunity against fatal Lassa fever, this monoclonal antibody cocktail has only been tested in non-human primates and rendered ineffective against the evolving Lassa virus lineage [302]. By 2023, CureVac AG Tübingen developed a prophylactic treatment for life-threatening Lassa fever using a non-viral LNP vector system [307]. Jasny (2023) created an mRNA-based vaccine encoding antigenic peptides expressing Lassa virus GPC and NP peptide, showing efficient responses at low doses. These inventors further demonstrated how mRNA-based vaccines can be engineered to encode different antigenic peptides expressing Lassa virus GPC precursor and NP peptide (from the highly evolving Lassa virus of clade I), showing efficient antigen-specific responses even at low dose regimen [307]. The therapeutic efficacy of the invented LASV-specific mRNA vaccine carrying a combination of antigenic peptides was evaluated in HeLa cells and mice models (balb/c) and induced robust T-cell and memory B-cell responses, with minimal side effects [307]. The current invention holds many promises and has now progressed to a phase I clinical trial. In a cohort study, the safety and efficacy profile of the LASV-specific mRNA vaccine was evaluated in human subjects following an intramuscular injection, receiving a prime-boost of 2 doses to complete the vaccination [307].

### mRNA based anti-cancer vaccines

Cancer vaccines include preventative vaccines to impede cancer development in healthy individuals and therapeutic vaccines to treat existing cancer by enhancing immune responses. RNA vaccines, particularly mRNA polynucleotides, have superior design features for protein translation by utilizing existing cellular machinery. Unlike conventional vaccines produced externally, RNA vaccines are administered in a manner resembling the body’s natural state. Valiante et al., patented a cancer vaccine using mRNA that encodes multiple cancer antigens and an immune checkpoint modulator. The vaccine involves administering RNA polynucleotides containing an ORF encoding antigenic polypeptides, inducing specific immune responses comparable to vaccination [308].

#### Lung cancer therapies

Genomic alterations in lung cancer affect both oncogenes and tumour suppressor genes. Chromosomal rearrangements, mitotic recombination, point mutations, and epigenetic events like promoter methylation can inactivate tumour suppressor genes [309]. Key tumour suppressor genes in lung malignancies include CDKN2 (p16INK4a or MST1, 9p21), RB1 (13q14.11), TP53 (17p13.1), and several genes at 3p [310].

Kallen et al. developed a cancer vaccine containing six mRNAs with histone stem loops in 3′UTR regions, encoding different antigen [311]. These antigens - MAGE-C2, 5T4 (TPBG), MAGE-Ci, MUC1, NY-ESO-1 (CTAG1 B), and survivin (BIRC5) - induce adaptive immune responses in mammals. The vaccine includes at least one mRNA encoding antigen combinations and is designed to stimulate immune response for treating lung cancer, specifically NSCLC, and related disorders [311].

#### KRAS pathway-based cancer vaccines

Ashburn et al. designed an LNP-based mRNA cancer vaccine for use in human patients. The vaccine delivers two separate mRNAs: the first encodes a collection of peptide epitopes, while the second encodes epitopes specifically derived from common cancer hotspot mutations, such as KRAS G12 or G13. By incorporating both personalized antigens and shared cancer mutations, the approach aims to generate a stronger and broader immune response. This strategy is further integrated with anti-cancer immunotherapy to improve treatment outcomes [312].

Valiante et al. introduced concatemeric, or poly-epitope, mRNA cancer vaccines, in which a single mRNA construct encodes multiple cancer epitopes [313]. These vaccines are designed to target key mutations such as KRAS and p53 and can also incorporate immune stimulators or adjuvants like STING to boost immune responses. Packaged in LNPs, the vaccine may contain one or more mRNA molecules, each with an ORF encoding at least two peptide epitopes. The process begins with analyzing tumour biopsies to identify cancer-specific antigens and selecting immunogenic epitopes for inclusion in the mRNA construct. Once delivered intradermally, intramuscularly, or subcutaneously, the vaccine instructs the host’s cells to generate a wide spectrum of tumour-derived proteins or fragments, thereby promoting a strong and targeted anti-cancer immune response [313].

#### Personalized cancer vaccine

Zhong et al. revolutionized strategies for producing, administering vaccines, and formulating vaccine compositions [314]. The formulation of certain cancer vaccines incorporates a single mRNA with an open reading frame (ORF) that encodes fifteen peptide epitopes. This vaccine has the potential to facilitate the production of nearly any desired cancer protein or its segment through the body’s cellular mechanisms. The proposed protocol for treating a cancer patient involves analyzing a patient-derived sample to identify one or more personalized cancer antigens, which are then assessed for the anti-tumour efficacy of at least two peptide epitopes for each identified personalized cancer antigen. Once the epitopes have been identified, the cancer vaccine is developed [314].

Chen et al. developed a method to predict cancer patient response to MDM2 agonists (formulas I–III) by measuring mRNA expression of MDM2, along with a panel of genes such as CDKN2A, XPC, and BBC3 [315]. This biomarker-based approach helps identify patients likely to benefit from therapies targeting the MDM2–p53 interaction. The study assessed several biomarkers, including CDKN2A, TNFRSFOB, BBC3, CCNG1, TRIAP1, CDKN1A, MDM2, FDXR, DDB2, XPC, EDA2R, RPS27L, and BAX, comparing MDM2 mRNA expression in patients to reference levels from others with the same cancer type [315]. To support this, the authors used 281 human cancer cell lines (CELLO) derived from diverse tumour types and tissues. Genomic profiling revealed that 210 of these cell lines carried TP53 mutations, underscoring the importance of integrating biomarker analysis with targeted treatment strategies [315].

#### Prostate cancer

Prostate cells are vulnerable to mutations that promote uncontrolled proliferation, ultimately leading to prostate cancer. Several tumour-associated antigens—including Ep-CAM, HER-2, PSCA, PAP, PSMA, and PSA—are significantly overexpressed in prostate cancer cells compared to normal tissue, making them valuable therapeutic targets. Conventional treatments such as chemotherapy, hormone therapy, radiation, and proton therapy, whether used alone or in combination, often provide only limited benefit.

To overcome these challenges, Kallen et al. explored the use of mRNA-based vaccines designed to encode prostate cancer–associated antigens [316]. Key targets included PAP, MUC1, STEAP, PSA, PSMA, and PSCA, with the mRNAs administered either as a single formulation or as multiple constructs, each encoding one antigen. By inducing a strong adaptive immune response, this strategy aimed to enhance tumour recognition and elimination. The approach showed potential applicability across diverse prostate cancer subtypes, including adenocarcinoma, locally advanced disease, castration-resistant forms, and metastatic cancers [316].

#### Immune enhancer mRNA therapy

Huang et al. extracted mRNAs encoding polypeptides that enhance immune responses to specific antigens [317]. These antigens activate pathways like Type I interferon or NF-κB, and influence dendritic cell development and inflammatory responses. The mRNAs contain modified nucleobases and can augment immune reactions when co-administered with antigens against cancer or pathogens [317].

Frederick et al. developed therapeutic strategies for cancer management. mRNAs coding for cell-associated cytokines (IL-15, IL-12) and costimulatory molecules (OX40L) activate T cells and NK cells, showing anti-tumour efficacy in solid tumours and myeloid malignancies [318]. The combination of mRNAs encoding IL-15, IL-12, and OX40L enhances anti-tumour treatment by efficiently activating T cells and NK cells [318].

Frederick et al. also proposed anti-cancer mRNA-combined therapies, combining at least two combined polynucleotides (mRNAs) [318]. The polynucleotides are limited to: (i) a polynucleotide encoding IL23 as immune response primer; (ii) a polynucleotide encoding OX40L as immune response co-stimulatory signal; (iii) a polynucleotide encoding anti-CTLA-4 antibody as checkpoint inhibitor; or (iv) their combinations. The therapy aims to reduce tumor size or inhibit growth in patients through intra-tumoural injection of these combinations [319].

## Future perspectives in mRNA therapeutics

The landscape of mRNA therapeutics is evolving rapidly, and several directions appear particularly important for the years ahead. One area attracting considerable attention is the development of cancer vaccines. Instead of applying a “one-size-fits-all” approach, future strategies are likely to rely on tailoring vaccines to the mutational signatures of individual tumours. Such personalization has the potential to improve therapeutic benefit while limiting unwanted side effects, making vaccines a central component of precision oncology.

Another avenue of progress lies in the treatment of inherited metabolic and genetic disorders. Early work in conditions such as citrullinemia type II demonstrates that mRNA can act as a temporary source of missing proteins. With refinement, the same principle could be extended to a much wider group of disorders where protein replacement is a rational therapy. At the same time, cancer research is exploring how mRNA treatments might be combined with existing modalities—checkpoint inhibitors, adoptive cell therapies, or even conventional chemotherapy—to produce stronger and longer-lasting responses than any single treatment alone.

Underlying all of these possibilities is the critical issue of delivery. mRNA is inherently unstable and easily degraded, so the development of more sophisticated carriers remains a central challenge. Advances in lipid nanoparticles, polymer formulations, and naturally derived vesicles are beginning to address these hurdles, and continued progress here will be essential if mRNA therapies are to reach their full range of applications. Importantly, these innovations are opening opportunities beyond oncology, with infectious disease prevention, neurodegeneration, cardiovascular disease, and autoimmune disorders all emerging as promising targets.

Scientific progress must, however, be matched with social responsibility. Questions of equitable access, transparent regulation, and long-term safety monitoring will be decisive in shaping the public’s trust. Education of both healthcare professionals and patients will also be vital to ensure that the benefits of mRNA technology are widely understood and accepted.

Finally, no single group can achieve these advances in isolation. The field will benefit most from strong collaborations linking academic laboratories, clinicians, industry partners, and regulatory authorities. Such partnerships will accelerate discovery and help to ensure that effective therapies move swiftly from the bench to the bedside.

In conclusion, mRNA therapeutics represent a genuine shift in modern medicine. Continued scientific innovation, coupled with thoughtful attention to delivery, accessibility, and collaboration, holds the promise of transforming care across a wide spectrum of diseases.
